# The microbiome-derived antibacterial lugdunin acts as a cation ionophore in synergy with host peptides

**DOI:** 10.1128/mbio.00578-24

**Published:** 2024-08-12

**Authors:** Anne Berscheid, Jan Straetener, Nadine A. Schilling, Dominik Ruppelt, Martin C. Konnerth, Birgit Schittek, Bernhard Krismer, Andreas Peschel, Claudia Steinem, Stephanie Grond, Heike Brötz-Oesterhelt

**Affiliations:** 1 Interfaculty Institute of Microbiology and Infection Medicine, Microbial Bioactive Compounds, University of Tübingen, Tübingen, Germany; 2 Microbial Bioactive Compounds, University of Tübingen, German Centre for Infection Research (DZIF), Partner Site Tübingen, Tübingen, Germany; 3 Institute of Organic Chemistry, University of Tübingen, Tübingen, Germany; 4 Georg-August-Universität Göttingen, Institute of Organic and Biomolecular Chemistry, Göttingen, Germany; 5 Department of Dermatology, Division of Dermatooncology, University of Tübingen, Tübingen, Germany; 6 Interfaculty Institute of Microbiology and Infection Medicine, Infection Biology, University of Tübingen, Tübingen, Germany; 7 Microbial Bioactive Compounds, University of Tübingen, Cluster of Excellence EXC 2124—Controlling Microbes to Fight Infections, Tubingen, Germany; 8 Max-Planck-Institute for Dynamics and Self Organization, Göttingen, Germany; MedImmune, Gaithersburg, Maryland, USA

**Keywords:** *Staphylococcus aureus*, antibacterial agent, natural product, bacteriocin, mechanism of action, membrane potential, synergism, dermcidin, microbiome interaction

## Abstract

Lugdunin is a microbiome-derived antibacterial agent with good activity against Gram-positive pathogens *in vitro* and in animal models of nose colonization and skin infection. We have previously shown that lugdunin depletes bacterial energy resources by dissipating the membrane potential of *Staphylococcus aureus*. Here, we explored the mechanism of action of lugdunin in more detail and show that lugdunin quickly depolarizes cytoplasmic membranes of different bacterial species and acidifies the cytoplasm of *S. aureus* within minutes due to protonophore activity. Varying the salt species and concentrations in buffers revealed that not only protons are transported, and we demonstrate the binding of the monovalent cations K^+^, Na^+^, and Li^+^ to lugdunin. By comparing known ionophores with various ion transport mechanisms, we conclude that the ion selectivity of lugdunin largely resembles that of 15-mer linear peptide gramicidin A. Direct interference with the main bacterial metabolic pathways including DNA, RNA, protein, and cell wall biosyntheses can be excluded. The previously observed synergism of lugdunin with dermcidin-derived peptides such as DCD-1 in killing *S. aureus* is mechanistically based on potentiated membrane depolarization. We also found that lugdunin was active against certain eukaryotic cells, however strongly depending on the cell line and growth conditions. While adherent lung epithelial cell lines were almost unaffected, more sensitive cells showed dissipation of the mitochondrial membrane potential. Lugdunin seems specifically adapted to its natural environment in the respiratory tract. The ionophore mechanism is refractory to resistance development and benefits from synergy with host-derived antimicrobial peptides.

**IMPORTANCE:**

The vast majority of antimicrobial peptides produced by members of the microbiome target the bacterial cell envelope by many different mechanisms. These compounds and their producers have evolved side-by-side with their host and were constantly challenged by the host’s immune system. These molecules are optimized to be well tolerated at their physiological site of production, and their modes of action have proven efficient *in vivo*. Imbalancing the cellular ion homeostasis is a prominent mechanism among antibacterial natural products. For instance, over 120 naturally occurring polyether ionophores are known to date, and antimicrobial peptides with ionophore activity have also been detected in microbiomes. In this study, we elucidated the mechanism underlying the membrane potential-dissipating activity of the thiazolidine‐containing cycloheptapeptide lugdunin, the first member of the fibupeptides discovered in a commensal bacterium from the human nose, which is a promising future probiotic candidate that is not prone to resistance development.

## INTRODUCTION

The microbiome affects human health in many ways, i.e., the skin microbiota helps to protect us against invading pathogens and educates our immune system, but its dysbiosis is associated with acne, atopic dermatitis, and chronic wounds (1, 2); an altered gut microbiota is linked to metabolic pathologies such as type 2 diabetes and prediabetes, obesity, and cardio- and hepato-metabolic diseases (3); and gut bacteria can also modulate patient responses to cancer immunotherapy (4). Moreover, the microbiome is a rewarding source of new and uncharacterized compounds with antibacterial activity. Ribosomally synthesized and post-translationally modified peptides represent the largest group of antimicrobials identified from microbiome-related producer strains (5). While considerably rarer, microbiome-derived small molecules with antimicrobial properties that are not ribosomally synthesized have also been discovered, such as nonribosomal peptides (NRPs) and polyketides. Bacteriocins, a term initially attributed to unaltered or modified ribosomally synthesized antimicrobial proteins or peptides, now often also refer to nonribosomally synthesized antibacterial molecules produced by members of microbiome communities. They have important roles in shaping the microbiome, i.e., by either facilitating or preventing community invasion (6). Bacteriocins have also been discussed as potential alternatives to traditional antibiotics (6–8). Importantly, bacteriocins from the microbiome have evolved to be effective at the body sites where they are produced and may be promising as therapeutic agents in their natural environment.

A few years ago, we discovered lugdunin, the first NRP bacteriocin purified from the human nose microbiome. Produced by the human nasal and skin commensal *Staphylococcus lugdunensis*, it effectively eradicates *Staphylococcus aureus* from the nose and the skin. Moreover, lugdunin has immunomodulatory functions by inducing the expression of host-derived antimicrobial peptides (AMPs), such as human cathelicidin LL-37, and chemokines recruiting monocytes and neutrophils (9, 10). In addition, synergistic activity in killing *S. aureus* was observed when lugdunin was combined with LL-37 or DCD-1 and DCD-1L, the latter being peptide fragments of the host-derived AMP dermcidin. In a first mode of action analysis, we monitored the incorporation of radioactively labeled metabolic precursors into different cellular macromolecules and observed that lugdunin led to a parallel cessation of the syntheses of DNA, RNA, protein, and cell wall, suggesting a collapse of bacterial energy resources (9). In a follow-up study, we showed that lugdunin dissipates the membrane potential of *S. aureus* and can mediate the transport of protons across artificial model membranes *in vitro* (11).

In the current study, we explored the mechanism of action of lugdunin in more detail. We analyzed the effect of lugdunin on the bacterial membrane potential in a time-resolved manner and used fluorescence microscopy to visualize the positioning of cell division proteins that depend on a healthy membrane potential for correct localization. We employed several bioreporters to test for possible effects of lugdunin on different bacterial metabolic pathways. Following up on our previous observation with membrane vesicles, we investigated if lugdunin acts as a protonophore in live *S. aureus* cells and if other cations can also be transported. In all assays, we compared the activities of lugdunin to reference antibiotics with known mechanisms of action. Furthermore, we explored the reason for the synergism between lugdunin and dermcidin-derived peptides. In addition to our experiments with live *S. aureus* cells, we conducted *in vitro* assays to determine ion-binding preferences and follow transport processes into artificial membrane vesicles to further characterize the ion selectivity of lugdunin. Finally, we tested lugdunin on different eukaryotic cell lines and monitored its effect on mitochondria using fluorescence microscopy. The current study is a coherent account of how lugdunin affects growing bacterial and eukaryotic cells.

## RESULTS

### Lugdunin quickly dissipates the membrane potential of bacteria

**Fig 1:**
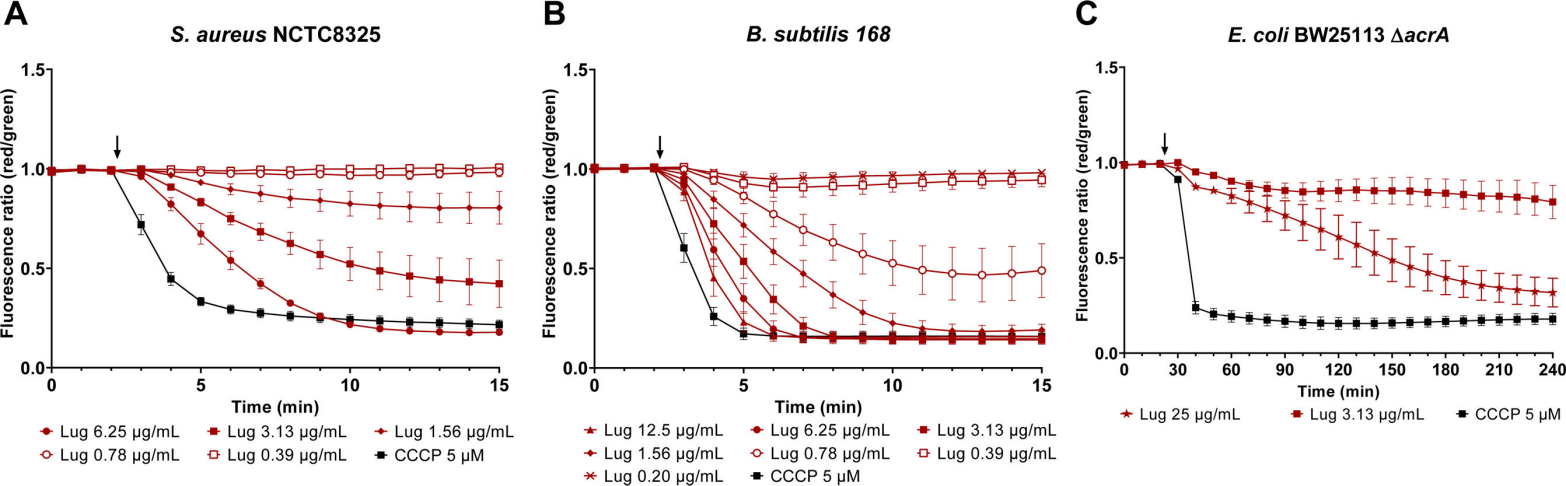
Lugdunin dissipates the membrane potential in different bacterial species. Upon lugdunin addition (black arrow), the cytoplasmic membranes of *S. aureus* NCTC8325 (**A**) and *B. subtilis* 168 (**B**) depolarize quickly and in a concentration-dependent manner. Lugdunin, albeit more slowly and at higher concentration, is able to dissipate the membrane potential of an efflux-deficient *E. coli* mutant (*E. coli* BW25113 ∆*acrA*) in the presence of the outer membrane permeabilizing agent PMBN (15 µg/mL) (**C**). The assays were conducted in phosphate-buffered saline (PBS) buffer with 0.1% glucose and 3,3′-diethyloxacarbocyanine iodide [DiOC_2_(3)] as the membrane potential probe. The highest lugdunin concentration shown corresponds to 2× MIC for each of the three strains. CCCP (5 µM) was used as the positive control. All data are normalized to an untreated DMSO control, which was set to a fluorescence ratio (red/green) of 1. The experiment was performed with at least three independent biological replicates with technical duplicates each. Error bars show the standard error of the mean (SEM).

We had previously observed that lugdunin leads to membrane depolarization in *Staphylococcus aureus* after 30 min of treatment. A strong correlation between the dissipation of the membrane potential and respective minimal inhibitory concentration (MIC) values of lugdunin and various derivatives had been noted (11). To explore the impact of lugdunin on bacterial membrane potential in more detail, we conducted time-resolved membrane depolarization assays in different bacterial species. Dissipation of the membrane potential in *S. aureus* and *Bacillus subtilis* occurred quickly (Fig. 1A and B). Within seconds after lugdunin addition, membranes started to depolarize, and complete depolarization comparable to the action of the reference protonophore carbonyl cyanide *m*-chlorophenylhydrazone (CCCP) was reached after 5–10 min in a concentration-dependent manner. Lugdunin-mediated membrane depolarization occurred faster in *B. subtilis* as compared to *S. aureus*. While lugdunin showed single-digit microgram per milliliter activity against various Gram-positive species including multi-resistant strains, it was mostly inactive against Gram-negatives (9). However, when we permeabilized the outer membrane of *Escherichia coli* using polymyxin B nonapeptide (PMBN) and used efflux-deficient mutants, we detected that lugdunin can inhibit the growth of Gram-negatives with compromised outer membranes, e.g., *E. coli* BW25113 ∆*tolC* MIC = 6.25 µg/mL (Table S1). Consistently, lugdunin dissipated the membrane potential of *E. coli* BW25113 ∆*acrA* when treated with PMBN (Fig. 1C), indicating that the missing bioactivity against wild-type Gram-negative bacteria is not due to the lack of a target but that the outer membrane forms a permeation barrier.

**Fig 2:**
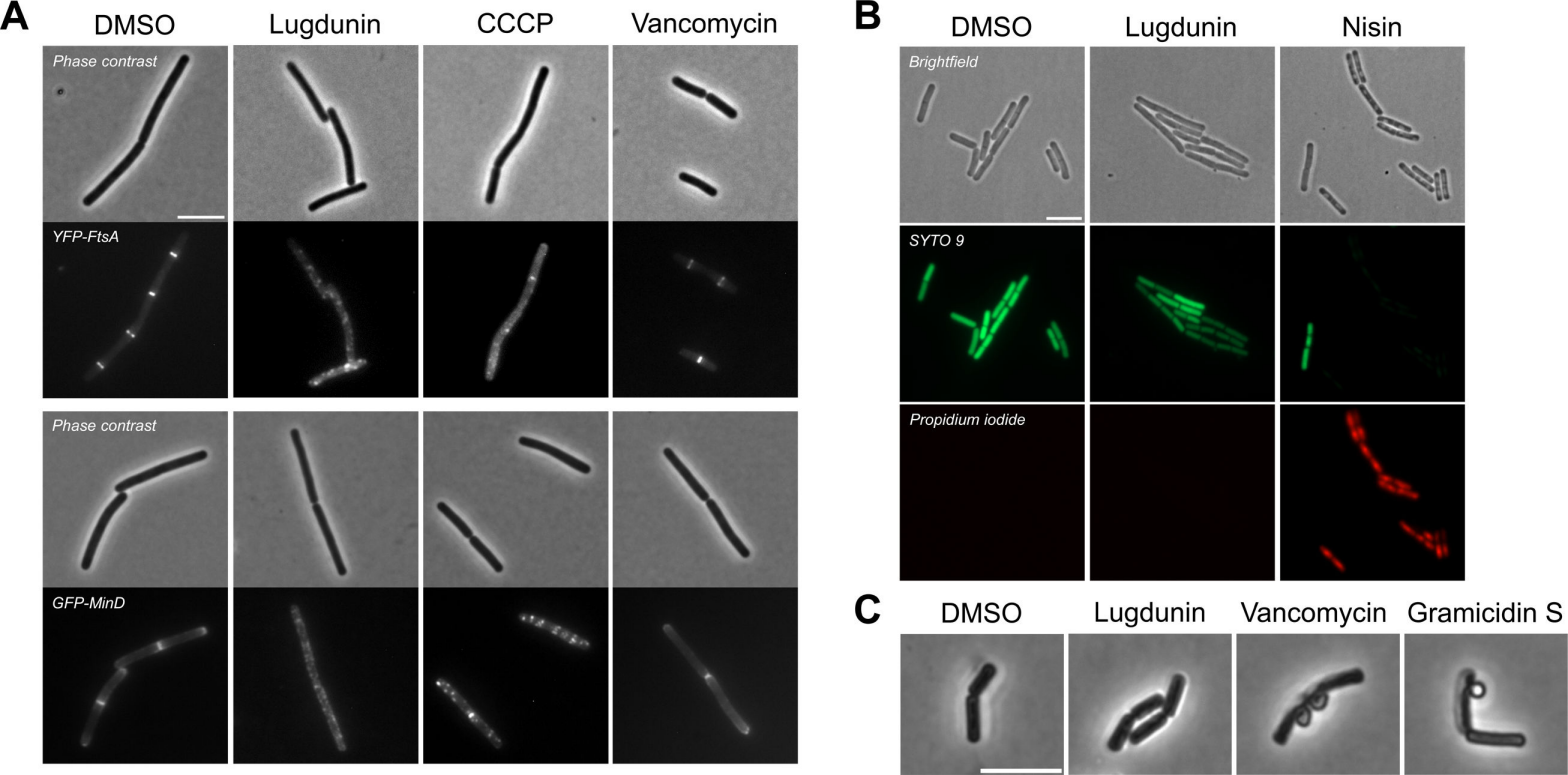
Fluorescence microscopy of lugdunin-treated *B. subtilis* 168 *trpC*2. (**A**) Lugdunin-mediated (10 µg/mL, 1.6× MIC) membrane depolarization caused delocalization of the cell division proteins YFP-FtsA and GFP-MinD similar to the positive control CCCP (50 µM, 5× MIC), while vancomycin (10 µg/mL, 20× MIC) served as a negative control. (**B**) Co-staining with the membrane-permeant SYTO 9 and the membrane-impermeant propidium iodide (PI) to monitor cytoplasmic membrane integrity. Both dyes emit fluorescence only after binding to DNA. Lugdunin (25 µg/mL, 4×MIC) did not lead to large membrane lesions or pore formation, as PI was not able to enter the cytoplasm. Crude nisin (100 µg/mL) was used as a positive control for the formation of pores. (**C**) Lugdunin (25 µg/mL, 4× MIC) does not cause formation of membrane blebs in *B. subtilis*. Membrane blebbing was observed after treatment with vancomycin (5 µg/mL, 10× MIC) or gramicidin S (12 µg/mL, 4× MIC). Scale bars, 5 µm.

The bacterial membrane potential has previously been described to be crucial for the proper localization of several peripheral membrane proteins involved in cell division, e.g., FtsA and MinD (12). Accordingly, fluorescence microscopy showed the delocalization of YFP-FtsA and GFP-MinD in *B. subtilis* upon treatment with 10 µg/mL (1.6× MIC) lugdunin for 20 min (Fig. 2A). The positive control CCCP caused a similar phenotype, while the addition of cell wall synthesis inhibiting vancomycin used as a negative control did not affect the localization of both cell division proteins in this time frame (Fig. 2A). Together with the observations that lugdunin led to a rapid and simultaneous cessation of incorporation of radioactive DNA, RNA, protein, or cell wall precursors (9), our data prove that lugdunin causes a quick depletion of energy resources in bacteria.

### Lugdunin does not cause large membrane lesions nor does it directly interfere with peptidoglycan synthesis

A common cause of quick membrane depolarization is the formation of membrane lesions, often called pores, that allow even the passage of molecules such as fluorescent dyes or ATP. However, in contrast to the known pore-forming lantibiotic nisin, we could not detect such membrane lesions in *B. subtilis* 168 *trpC2* exposed to lugdunin when co-staining the cells with the membrane-permeable dye SYTO 9 and the membrane-impermeable PI. PI could not enter the cells even after 30 min of exposure to 25 µg/mL (4× MIC) of lugdunin (Fig. 2B). We had made a similar observation for *S. aureus* treated with 10× MIC of lugdunin previously (11). The lack of PI entry is in line with the report that lugdunin was not able to lyse artificial 1-palmitoyl-2-oleoyl-*sn*-glycero-3-phosphocholine (POPC) membranes in a carboxyfluorescein leakage assay (11). In accordance, no quick lysis of *S. aureus* had been observed upon lugdunin treatment (10× MIC) because the compound showed only delayed bactericidal activity after 24 hours of treatment (9). Due to its site of action at the bacterial cell membrane, we further checked for potential effects of lugdunin on the cell wall, i.e., the peptidoglycan sacculus. Within our bioreporter panel in *B. subtilis*, two cell envelope stress-detecting biomarkers *ypuA* and *liaI* were not induced when treating the cells with lugdunin (Table S2). Both biomarkers were previously validated to be induced upon treatment with cell wall active compounds, with *ypuA* broadly signaling cell membrane and cell wall damage, while *liaI* prominently signals an interference with the synthesis or function of undecaprenyl phosphate containing cell wall precursors in the cytoplasmic membrane (13–15). Notably, both bioreporters do not detect ionophore activity. Additionally, we did not observe the formation of blebs when treating the cells with 25 µg/mL lugdunin (4× MIC) for 30 min followed by a fixation procedure with acetic acid/methanol. Such blebs occur when the cytoplasmic membrane protrudes through breaches in the peptidoglycan sacculus and were clearly triggered by the reference compounds vancomycin and gramicidin S, which affect peptidoglycan integrity (Fig. 2C).

To exclude interference with other bacterial metabolic pathways, we performed reporter assays with our additional, previously well-characterized biomarkers (13, 14). We did not detect an induction of *yorB* (DNA stress), *helD* (RNA stress), nor *bmrC* (translational stalling) upon lugdunin treatment (Table S2). In a cell-free *in vitro* transcription/translation assay based on *E. coli* S30 extracts (16), lugdunin did not inhibit the expression of the luciferase reporter enzyme (Fig. S2). All data concur with the notion that lugdunin-mediated bacterial death is based on rapid dissipation of the membrane potential and not on direct interference with a specific metabolic pathway.

### Lugdunin shows protonophore activity in *S. aureus*

**Fig 3:**
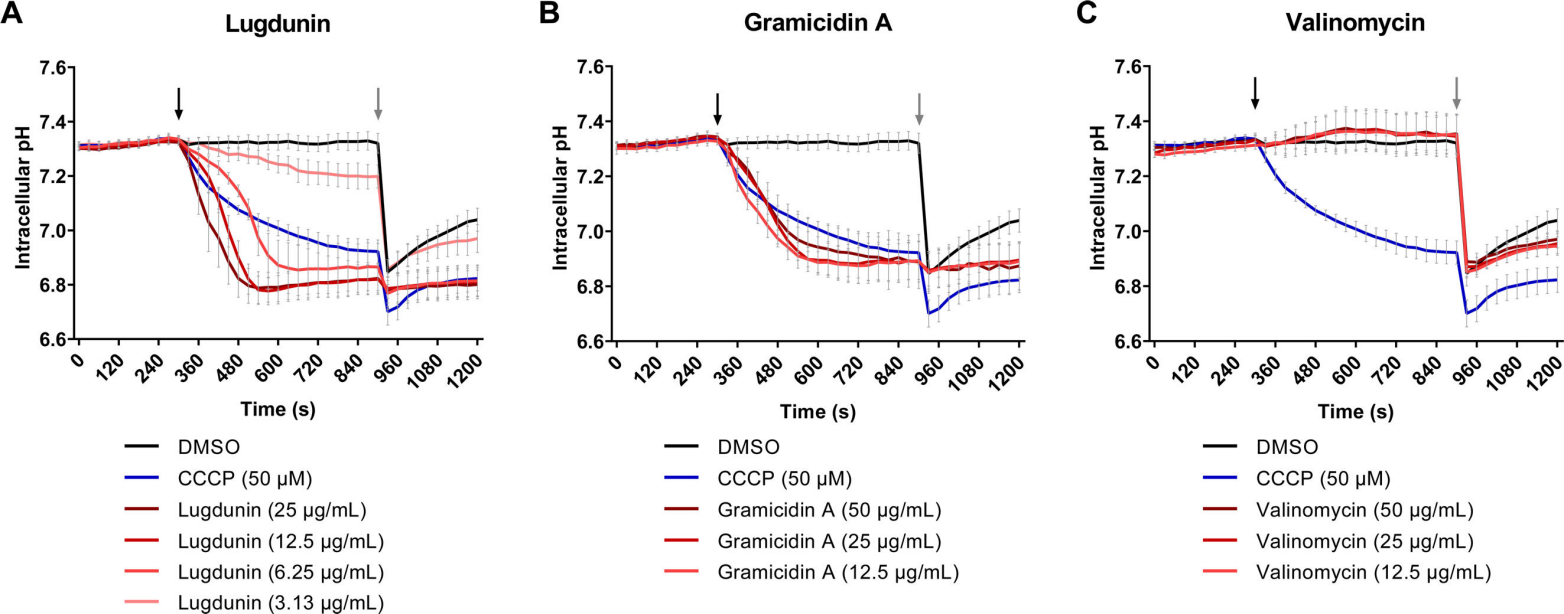
Lugdunin shows protonophore activity in *S. aureus* cells. In *S. aureus* NCTC8325 cells loaded with the pH-sensitive dye BCECF, lugdunin caused acidification of the cytoplasm in a concentration-dependent manner (**A**). Acidification was also observed upon the addition of gramicidin A (**B**), but not valinomycin (**C**). Concentrations used correspond to 1–8× MIC for lugdunin (3.13–25 µg/mL), 0.6–2.5× MIC for gramicidin A (12.5–50 µg/mL), and 0.6–2.5× MIC for valinomycin (12.5–50 µg/mL). The protonophore CCCP (50 µM, 5× MIC) was used side-by-side as a positive control in all experiments. The addition of the compounds is indicated by a black arrow. Ten minutes after compound addition, nigericin (20 µM) was added to all samples (gray arrow) as a control that allows quick acidification of the cytoplasm. The experiment was performed with at least four independent biological replicates with technical duplicates each. Error bars show the SEM.

Since pore formation, which would also lead to leakage of ions thereby dissipating the membrane potential, does not occur (Fig. 2B) (11), we postulated that lugdunin exhibits a more specific ionophoric activity and used the intracellular fluorescent pH probe 2′,7′-bis-(2-carboxyethyl)-5-(6)-carboxyfluorescein acetoxymethyl ester (BCECF-AM) to monitor pH changes in the cytoplasm of *S. aureus*. The established protonophore CCCP served as a positive control. Within seconds after the addition of lugdunin or CCCP, the *S. aureus* cytoplasm showed acidification (Fig. 3A). When we compared further ionophores in the same assay, it turned out that the ion channel-forming peptide gramicidin A also led to a drop in the cytoplasmic pH, while potassium-selective valinomycin did not (Fig. 3B and C). The data show that lugdunin exerts protonophore activity on bacterial cells, comparable to CCCP and gramicidin A.

### Membrane depolarization caused by lugdunin is salt-dependent

**Fig 4:**
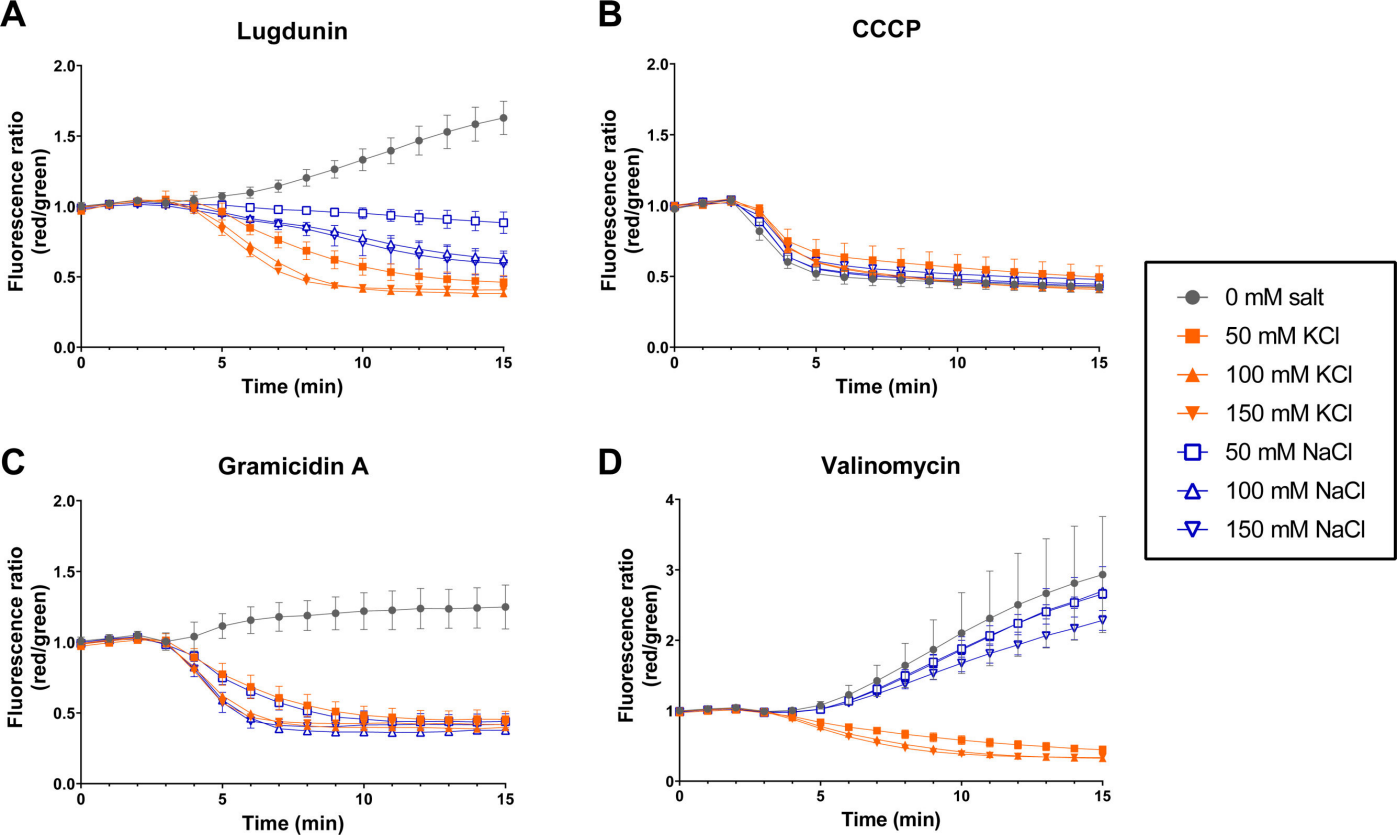
Dissipation of the membrane potential is affected by different salt species and salt concentrations. Lugdunin (3 µg/mL, 3.83 µM, 1× MIC) treatment led to membrane depolarization of *S. aureus* NCTC8325 in a 50 mM Tris buffer containing different concentrations of NaCl and KCl, while hyperpolarization was observed when no additional salts were present (**A**). Depolarization caused by lugdunin was stronger with KCl than NaCl. Contrarily, CCCP (5 µM, 0.5× MIC) led to depolarization under all buffer conditions (**B**). Gramicidin A (5 µM, 0.5× MIC) caused depolarization in the presence of NaCl and KCl and slight hyperpolarization in the absence of salts (**C**). Valinomycin (5 µM, 0.25× MIC) caused membrane depolarization only when KCl was added to the Tris buffer, while hyperpolarization occurred without added salts as well as in the presence of NaCl (**D**). DiOC_2_(3) was used as a membrane potential probe in all assays. All data were normalized to the measured 0 min value of the DMSO control, which was set to a fluorescence ratio (red/green) of 1. The experiment was performed with two independent biological replicates (different cultures on different days) with technical duplicates (two parallel aliquots each day). Error bars show the standard deviation (SD).

Activity and behavior of ionophores can be dependent on experimental conditions, e.g., compositions of the media or buffer surrounding the bacterial cells. To characterize the ionic behavior of lugdunin, we compared its ability to affect membrane potential to several well-characterized ionophores (compound structures depicted in Fig. S1) under different buffer conditions. Of note, in the membrane potential measurements shown in Fig. 1, PBS buffer was used, which by default contains sodium and potassium, and glucose had been offered, resulting in well-energized cells. In the following experimental series, we tested for a potential ion preference of lugdunin by varying the ion species and concentrations, changing the pH, and exploring the impact of glucose as an energy source. For comparison, we employed the proton-specific carrier ionophore CCCP, the potassium-specific carrier ionophore valinomycin, and the less selective channel-forming ionophore gramicidin A, which allows the translocation of different monovalent cations. In Tris buffer at pH 7.1 without the addition of salt ions or glucose, lugdunin, gramicidin A, and valinomycin led to hyperpolarization of *S. aureus*, which means that membrane potential was raised (more strongly negative inside). Only CCCP depolarized the cytoplasmic membrane and reduced the intracellular negative charge in salt-free buffer, and it was unaffected by the addition of salts (Fig. 4). In accordance with its proton selectivity, only the proton motive force influenced the transport rate of CCCP. Adding NaCl in different concentrations to the Tris buffer allowed lugdunin and gramicidin A to also cause depolarization, implying an interaction with sodium, while valinomycin, which has a very low affinity for sodium ions and is very selective for potassium ions within the cell membrane, still yielded hyperpolarization (Fig. 4). Finally, KCl addition triggered membrane depolarization in the presence of lugdunin, gramicidin A, and valinomycin (Fig. 4). Concluding, the reference ionophores behaved as expected, and lugdunin demonstrated a capacity to interact with K^+^ and Na^+^ (besides its previously demonstrated ability to transport protons). Of note, lugdunin-mediated depolarization was stronger in the presence of KCl compared to NaCl at equal salt concentrations. This selectivity trend was not observed for gramicidin A.

The assays presented in Fig. 4 were conducted in buffer without glucose. When 0.1% glucose was added to the Tris buffer, cells were better energized at the start of the experiment, and untreated cells had higher membrane potentials than cells in buffer without glucose. The higher charge difference (more negative at the inner leaflet of the cytoplasmic membrane) that is reflected by the higher membrane potential facilitates the entry of cations. Interestingly, ionophore action strongly reduced the differences between the initial membrane polarization levels (+/− glucose) toward an ionophore-specific level of the membrane potential (Fig. S3). The specific height of the plateau of the membrane potential reached after equilibration of the transmembrane ion gradients by a given ionophore seems to depend on the ion selectivity of the ionophore and the applied extracellular buffer conditions (i.e., ion species and concentration and glucose). Lugdunin again showed parallels with gramicidin A in this experiment, and there were clear differences to CCCP or valinomycin. Also, the pH of the surrounding buffer influenced the behavior of the ionophores differently. While at lower extracellular pH, lugdunin and gramicidin A treatment resulted in hyperpolarization of the cells in the absence of salts; both compounds led to salt-independent membrane depolarization at a more alkaline pH (Fig. S4). Valinomycin always caused hyperpolarization, and CCCP always caused depolarization in the tested pH range from 6.5 to 8.5 (Fig. S4).

Taken together, the effects of lugdunin in the membrane potential assays performed under varied buffer conditions most closely resembled those of gramicidin A and substantially differed from those of the single ion-selective ionophores valinomycin and CCCP. These data imply that lugdunin does not only have protonophore activity in *S. aureus* but also additionally transports further monovalent cations, such as K^+^ and Na^+^. Valinomycin or CCCP proved more ion-selective than lugdunin.

### Lugdunin binds and transports different monovalent cations

**Fig 5:**
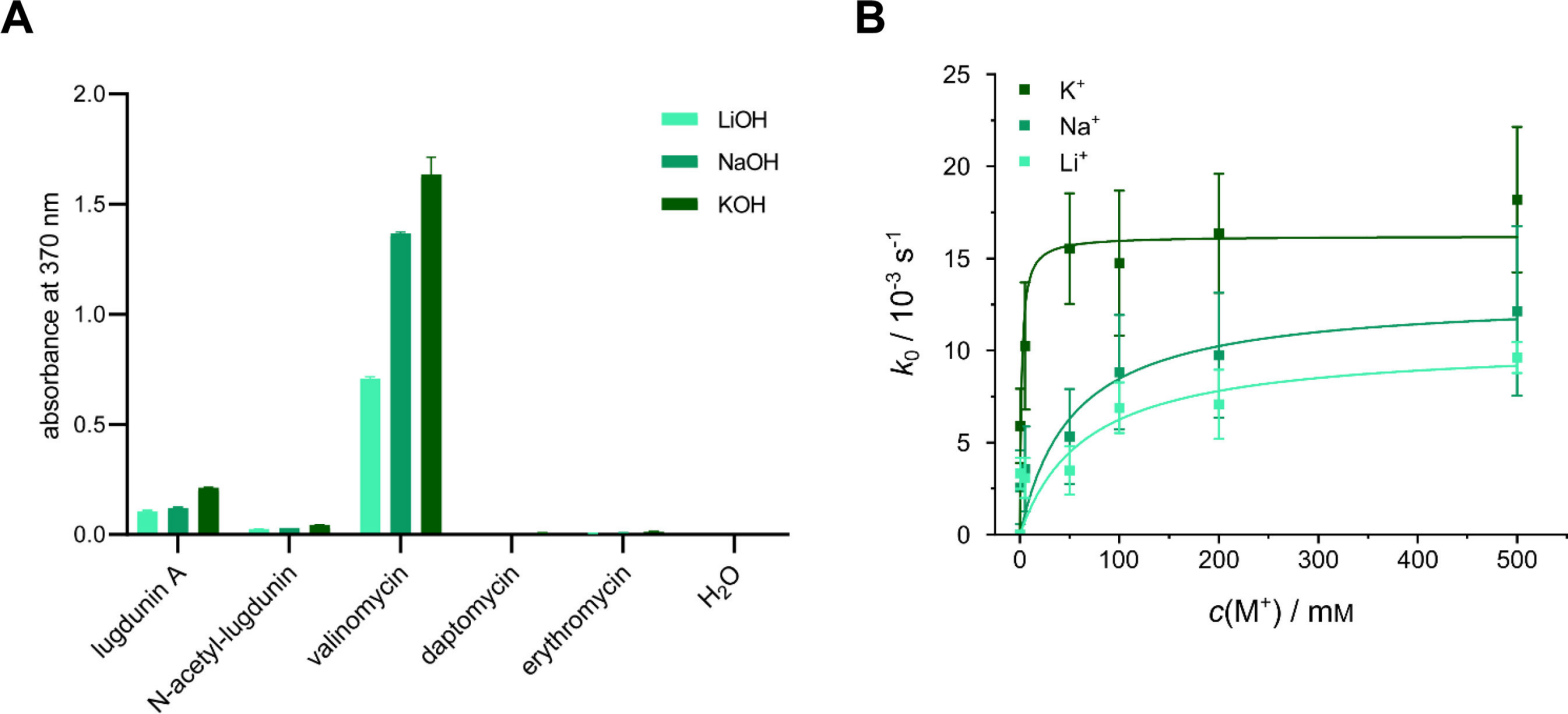
*In vitro* assays show binding and transport of monovalent cations by lugdunin. (**A**) The picric acid assay shows binding of lugdunin to Li^+^, Na^+^, and K^+^ ions. The derivative *N*-acetyl-lugdunin with strongly decreased antimicrobial activity (MIC *S. aureus* NCTC8325 ≥100 µg/mL) showed reduced binding of the respective monovalent ions. The ionophore valinomycin strongly interacted with all of the three tested monovalent ions. For daptomycin and erythromycin, no significant interaction was detected under these conditions. H_2_O was employed as a negative control. The assay was performed with three replicates; error bars show the SD. (**B**) Proton transport rates into artificial phospholipid vesicles obtained from the normalized initial slopes of fluorescence time traces (compare Fig. S6) depending on the salt concentration. Lugdunin was added to POPC vesicles filled with the pH-sensitive dye pyranine (peptide-to-lipid ratio 1:250) and exposed to a pH gradient (pH_in_ = 7.4, pH_out_ = 6.4). At the start of each experiment, salt concentrations (ranging from 0 to 500 mM KCl, NaCl, or LiCl) were equal between the lumen of the vesicles and the surrounding buffer. From fitting equation E1 (see Fig. S6) to the data, the transport constant *K*_T_ was determined reporting on the transport affinity of lugdunin for K^+^, Na^+^, and Li^+^. Data represent the mean ± SEM from greater than or equal to five measurements per salt concentration.

The ion-binding capacity of lugdunin was also analyzed *in vitro* in an assay using the indicator agent picric acid. The assay is based on alkali metal picrates (17, 18), which are insoluble in organic solvents but can be extracted from aqueous solution into the hydrophobic organic phase when in solution with a competing ionophore that binds monovalent alkali cations. The released picrate anion is brightly yellow-colored, which allows for spectrophotometric determination. Here, we observed moderate binding of the monovalent cations potassium, sodium, and lithium by lugdunin in the nonpolar dichloromethane (CH_2_Cl_2_) environment as a model surrounding in this assay (Fig. 5A). A strong binding of these ions *in vitro* was observed for valinomycin, in accordance with previous studies (19–21). The antibiotics daptomycin and erythromycin did not show any or only very low levels of binding under the same assay conditions (Fig. 5A). Interestingly, the lugdunin derivative *N*-acetyl-lugdunin (Fig. S1) displayed only very poor binding of these monovalent ions, in accordance with a strongly reduced ability to depolarize *S. aureus* membranes (Fig. S5A) and decreased antimicrobial activity (MIC ≥100 µg/mL). Also for other lugdunin analogs, a strong correlation between antibacterial potency and the capacity to dissipate the electrochemical gradient across the bacterial membrane had been observed (11).

Experiments using a membrane model system corroborate that lugdunin does not solely act as a protonophore but can also facilitate the transport of different monovalent cations. We hypothesized previously that metal ions might be transported by lugdunin in response to proton influx into vesicles to maintain a charge equilibrium between the intra- and extravesicular space (11). Here, we employed large unilamellar vesicles (LUVs) composed of palmitoyl-oleoyl phosphatidylcholine (POPC) filled with the pH-sensitive dye pyranine and exposed them to a pH gradient to study the proton transport capability of lugdunin in the absence or presence of the ions K^+^, Na^+^, and Li^+^. Of note, while we tested the impact of a salt concentration range (0–500 mM KCl, NaCl, or LiCl), the initial intra- and extravesicular salt concentrations were equal in each individual experiment. In the absence of monovalent cations (0 mM salt), proton influx into the vesicle lumen was greatly impeded as a result of the build-up of an electrochemical gradient (Fig. S6). In contrast, in the presence of salt, proton influx along the pH gradient was prominent and fast as measured by fluorescence quenching (Fig. S6), which let us conclude that a concurrent metal ion transport takes place leading to charge equilibration. Following this idea, monitoring the proton transport rate can be used as an indirect measure of metal ion transport. We quantified the proton transport rate as the initial linear slope *k*_0_ of the normalized fluorescence time curve as a function of different concentrations of KCl, NaCl, and LiCl, respectively (Fig. 5B). For all cations, the resulting curves saturate at high salt concentrations. In previous studies, such translocation processes, either carrier- or noncarrier-based, have been formally considered to be analogous to an enzymatic reaction validating the use of a Michaelis–Menten-like kinetic (22). For simplicity, we assume that for each translocated proton into the vesicle lumen, one metal ion is transported out of the vesicle, and thus, no transmembrane potential is generated. For this simple scenario, equation E1 (Fig. S6) is valid (23). Fitting equation E1 to the data allows a quantitative evaluation of the transport kinetics as a function of the cations K^+^, Na^+^, and Li^+^ (Fig. 5B). The transport constant *K*_T_ reports on the transport affinity of lugdunin for the corresponding cation. Similar to the Michaelis–Menten constant *K*_M_, *K*_T_ denotes the salt concentration at the half-maximum transport rate. Smaller *K*_T_ values correspond to a higher transport affinity. For K^+^, *K*_T_ = 1.6 ± 0.8 mM was obtained, whereas *K*_T_ = 53 ± 31 mM was found for Na^+^, and *K*_T_ = 66 ± 59 mM for Li^+^. The smaller *K*_T_ value for K^+^ demonstrates a higher affinity of lugdunin for potassium ions compared to sodium and lithium ions. Even though the differences between sodium and lithium ion translocation are not significant due to the low sensitivity of the assay for small *k*_0_-values, the general trend is in line with the findings presented in the picric acid assay (Fig. 5A) and with results from single-channel recordings of lugdunin in black lipid membranes (24).

### Synergism of lugdunin and dermcidin occurs on the level of membrane depolarization

In addition to its antibacterial activity, lugdunin has immunomodulatory functions by inducing the production of pro-inflammatory cytokines in keratinocytes (10). Lugdunin also induced the expression of AMPs like LL-37, thereby further amplifying innate immune responses in the skin. Interestingly, it was noted in a previous study that lugdunin acts synergistically with AMPs in killing *S. aureus* as shown by a calculated combination index <1, and the simultaneous presence of the compounds was shown to be necessary for synergistic action (10). Besides LL-37, a synergism of the AMP dermcidin and lugdunin is notable, as dermcidin and its proteolytically processed antimicrobially active peptides (DCD-1/DCD-1L) are constitutively expressed in eccrine sweat glands and transported to the skin surface, which is also the natural habitat of the commensal *S. lugdunensis* (10).

**Fig 6:**
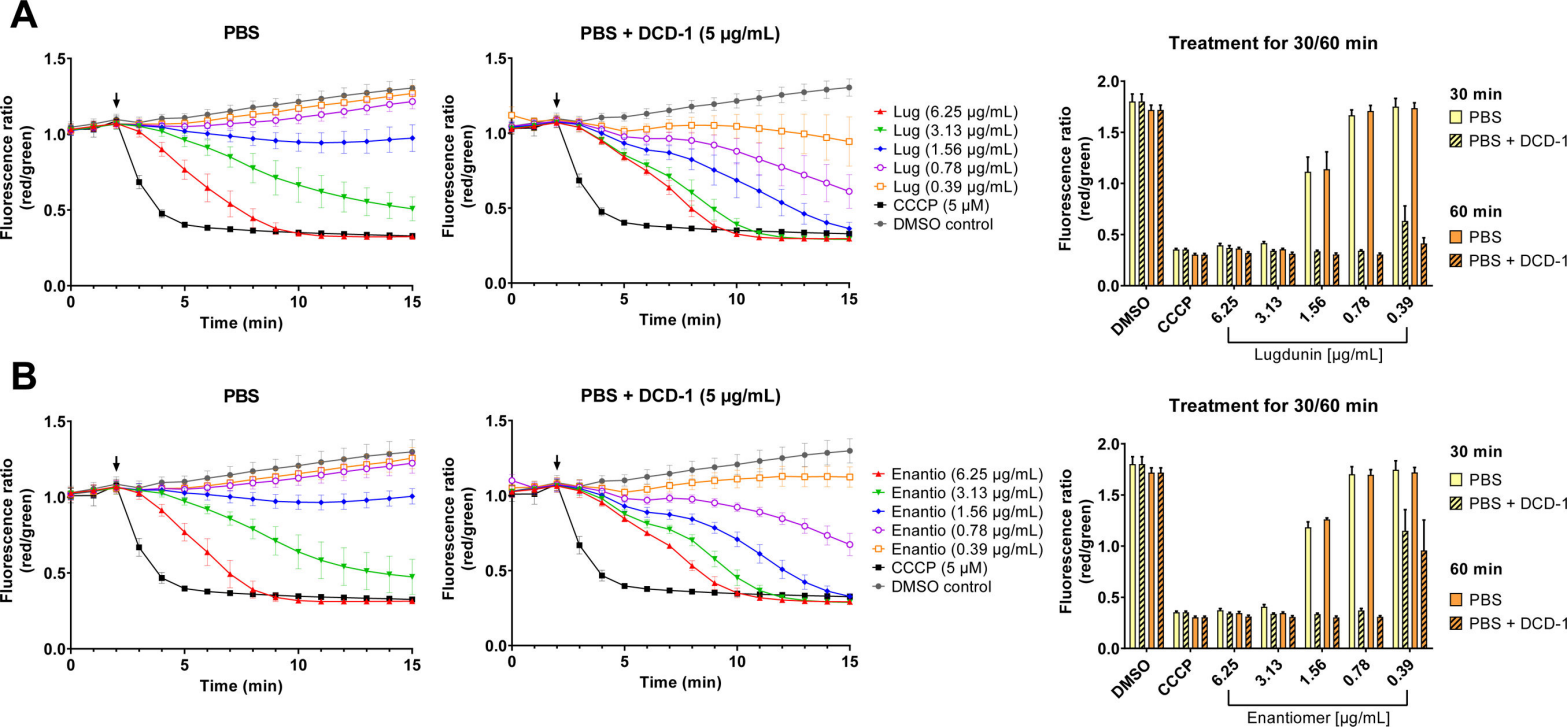
Synergistic action of lugdunin and its enantiomer with dermcidin-derived peptide DCD-1. In membrane potential assays with DiOC_2_(3), the addition of 5 µg/mL DCD-1 lowered the concentration of lugdunin (**A**) or its enantiomer (**B**) necessary to cause membrane depolarization of *S. aureus* NCTC8325 by more than fivefold. The synergistic effect emerged immediately after compound addition (black arrows) and established itself prominently and stable (compare right panels after 30 and 60 min of compound exposure). The concentration of DCD-1 (5 µg/mL) employed in the assay does not cause membrane depolarization by itself. The experiment was performed in PBS buffer with at least three biological replicates with technical duplicates each. Error bars show the SEM.

Here, we examined the synergistic effect of lugdunin and dermcidin in a mechanistic manner. When combining lugdunin and dermcidin-derived peptide DCD-1 in membrane potential experiments with *S. aureus*, we observed a concerted action of the two peptides at the level of membrane depolarization. When treating the cells with DCD-1 at a concentration (5 µg/mL) that did not affect the membrane potential by itself, the combination with lugdunin led to enhanced dissipation of the membrane potential compared to the activity of lugdunin alone (Fig. 6). Likewise, low sub-MIC concentrations of lugdunin (e.g., 0.78 and 0.39 µg/mL), which alone did not lead to membrane depolarization, were able to dissipate the *S. aureus* membrane potential when combined with 5 µg/mL of DCD-1 (Fig. 6A). The same observation was made using a combination of lugdunin and DCD-1L, another naturally occurring dermcidin variant containing an additional leucine (Fig. S5B). Importantly, concerted action was also observed for the enantiomer of lugdunin in combination with DCD-1 (Fig. 6B).

Co-staining of *S. aureus* with the membrane-permeant SYTO 9 and membrane-impermeant PI demonstrated that the amount of DCD-1 employed in the membrane potential assay (5 µg/mL) did not cause marked membrane lesions or larger pores, and neither did the combination treatment of lugdunin at 4× MIC (12.5 µg/mL) and DCD-1 (5 µg/mL) (Fig. S7), implying that the observed effect on the bacterial membrane potential is not based on general membrane disruptions but more specifically caused by an increased membrane permeability to ions.

### Lugdunin can affect certain eukaryotic cells depending on cell line and growth condition

**Fig 7:**
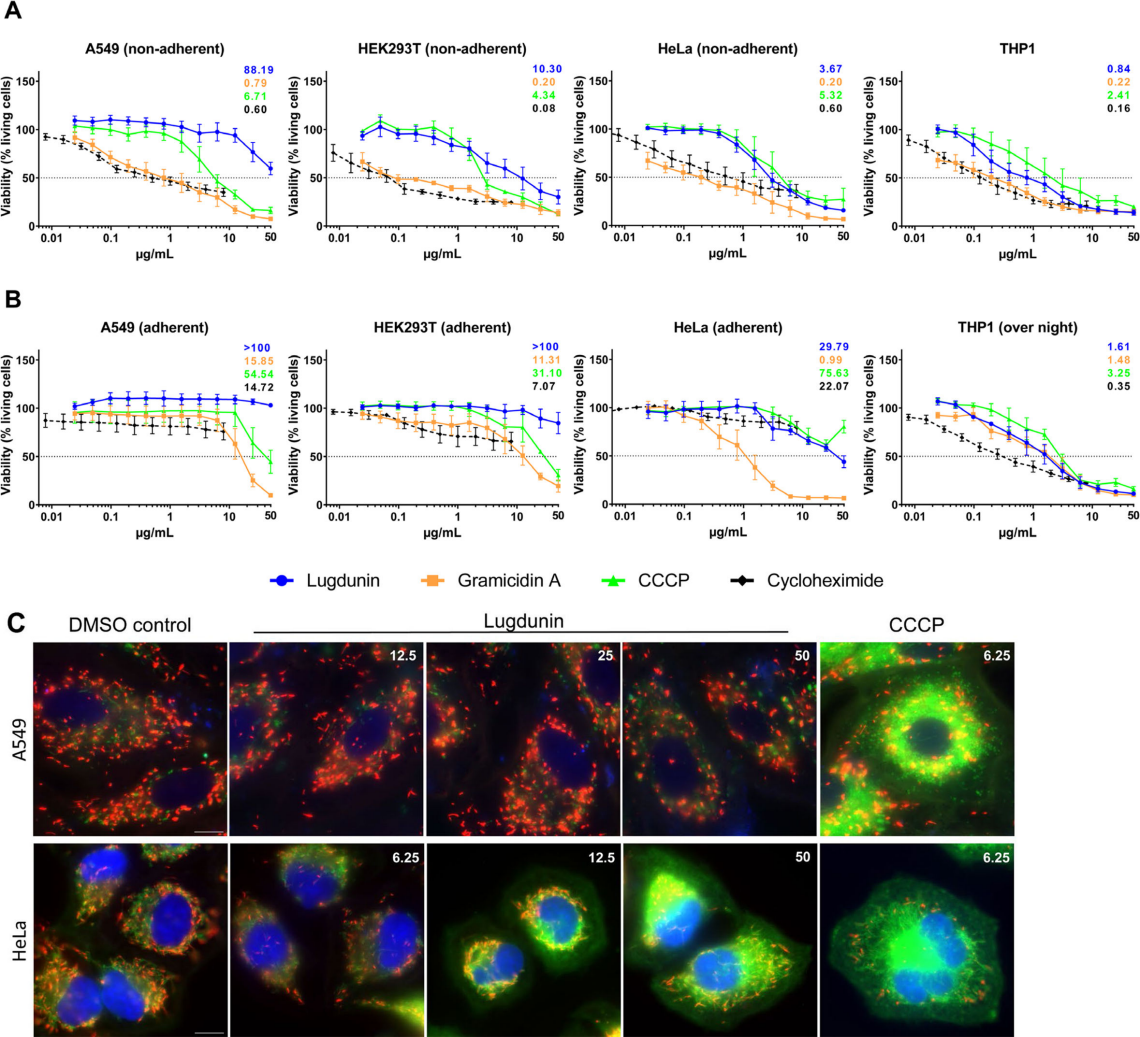
Activity of lugdunin on eukaryotic cells is strongly cell line- and growth condition-dependent. (**A**) When treated with compounds immediately after seeding, the epithelial cell lines A549 and HEK293T were less susceptible to lugdunin than the suspension cell line THP-1. The ionophore gramicidin A showed notably higher cytotoxic activity against all tested cell lines. (**B**) When the cells were allowed to settle overnight before treatment, the adherent epithelial cell lines A549, HEK293T, and HeLa were increasingly resistant to lugdunin, while THP-1 cells remained susceptible. Half-maximal inhibitory concentrations (IC_50_) were calculated by nonlinear regression in GraphPad Prism. IC_50_ values (in micrograms per milliliter) are depicted for each cell line in their respective graph, and colors reflect the compound used for treatment. Cell viability assays were performed with minimum of three biological replicates; error bars represent the SEM. (**C**) Mitochondrial membrane potential of A549 and HeLa cells after compound treatment for 10–20 min was visualized using the JC-1 dye. Mitochondria of A549 cells appeared largely unaffected up to the tested concentration of 50 µg/mL. The observed depolarization of mitochondria in HeLa was notable from 12.5 µg/mL. CCCP (6.25 µg/mL) was used as a positive control to depolarize mitochondrial membranes. Numbers in white in the pictures represent the respective compound concentrations in micrograms per milliliter. Scale bars, 10 µm.

Ionophores are molecules that contain a hydrophobic moiety enabling them to insert into or cross biological membranes. As some ionophores have rather unspecific membrane affinity, they often also show activity against eukaryotic cells. To evaluate this potential risk for lugdunin, we analyzed its activity against different mammalian cell lines under two different environmental conditions. Eukaryotic cells were exposed to lugdunin and reference compounds either immediately after seeding into a new cell culture plate (i.e., in a nonadherent, nonconfluent state) or after settling overnight before treatment. It should be noted that for epithelial cells, the freshly trypsinized, nonadherent, nonconfluent state diverts substantially from their physiological condition. The epithelial cell lines A549 (human lung adenocarcinoma) and HEK293T (human embryonic kidney) were less susceptible to lugdunin compared to the suspension cell line THP-1 (human leukemia monocytes), and this difference was particularly strong after the epithelial cells had settled and adhered (Fig. 7A and B). Gramicidin A inhibited cell viability much more strongly than lugdunin.

Moreover, we analyzed lugdunin-treated A549 and HeLa (human cervix adenocarcinoma) cells by performing fluorescence microscopy with JC-1, a cationic lipophilic dye that shows a membrane potential-dependent accumulation in mitochondria, with a detectable red emission (~590 nm) of the red fluorescent aggregates (J-aggregates), which form at healthy membrane potentials, and a shift to green fluorescence emission (~529 nm) for the monomeric form occurring after membrane potential dissipation. Mitochondria of A549 appeared unaffected up to the highest tested lugdunin concentrations of 50 µg/mL, consistent with their high IC_50_ for lugdunin. For HeLa, we observed a concentration-dependent depolarization of mitochondrial membranes after 10–20 min of lugdunin treatment (Fig. 7C). While most of the cells were unaffected at 6.25 µg/mL lugdunin, more cells showed dissipation of the mitochondrial membrane potential at 12.5 µg/mL, indicating that lugdunin has also the ability to affect the ion gradient across the inner mitochondrial membrane of sensitive cells.

## DISCUSSION

We had previously observed that lugdunin causes a simultaneous breakdown of the main bacterial metabolic pathways, indicating rapid depletion of energy resources (9). In addition, lugdunin dissipated the membrane potential of *S. aureus* in a concentration-dependent manner, and its derivatives demonstrated a strong correlation between the strength of this activity and their MIC (11). In our current study, we investigated the mechanism of action of lugdunin in depth. Membrane depolarization started immediately upon lugdunin addition to bacterial cells and was discernable already at concentrations below the MIC, implying that membrane de-energization is an important and primary consequence of lugdunin’s action. Our data converge on the conclusion that lugdunin renders the cytoplasmic membrane leaky for ions. Consistently, the cell division proteins FtsA and MinD (Fig. 2A), which rely on the transmembrane potential for proper localization (12), delocalized in the presence of lugdunin. This protein delocalization is a hint that the flux of ions promoted by lugdunin, like the positive control for this assay, CCCP, is electrogenic, i.e., changing the charge distribution across the cytoplasmic membrane. In contrast, nigericin, a polyether antibiotic that primarily promotes an electroneutral exchange of H^+^ and K^+^ ions (25), did not cause a delocalization of FtsA and MinD (12). We can exclude that lugdunin disrupts membrane integrity by causing a pore in the cytoplasmic membrane of *B. subtilis* (Fig. 2B) or *S. aureus* (11), as lugdunin, in contrast to the reference pore former nisin (26), did not allow permeation of dye molecules.

To not overlook potential additional effects on specific metabolic pathways, we tested a panel of bioreporter strains, which had been extensively validated previously using a large set of antibiotics (13, 14). Lugdunin did not induce the expression of the biomarkers *ypuA* and *liaI*, known to signal various kinds of cell envelope stress but not ionophore activity. Consistently, those promoters were also not induced by our reference ionophores CCCP, gramicidin A, and valinomycin, while antibiotics with known targets in bacterial cell wall synthesis, like nisin, vancomycin, and daptomycin, triggered their increased expression (Table S2). Our bioreporter data are in accordance with proteome data, where the ionophores CCCP, gramicidin A, and valinomycin did not induce LiaH expression (27). Congruent is also the result that lugdunin did not induce membrane blebbing, a phenotype associated with breaches in the peptidoglycan sacculus, i.e., as caused by vancomycin (Fig. 2C). Of note, gramicidin S (Fig. S1), a cyclic antimicrobial peptide consisting of 10 amino acids and no ionophore activity despite its similarity of name to gramicidin A, quickly dissipates the bacterial membrane potential but also induces the expression of the *liaI/*LiaH biomarkers and causes bleb formation, showing that its mechanism of action includes an interference with cell envelope synthesis (Fig. 2C; Table S2) (11, 28, 29). For lugdunin, we further excluded a direct interference with DNA, RNA, and protein synthesis by bioreporter assays and *in vitro* transcription translation assays. Altogether, our mode of action profiling concurs that lugdunin acts as an ionophore on bacterial cytoplasmic membranes without directly impacting cell wall/envelope biosynthesis. The observation that the enantiomer of lugdunin has the same membrane potential-dissipating capacity as native lugdunin (Fig. 6) (11) confirms the lack of a chiral protein or peptidoglycan binding partner and provides an explanation for the previously reported lack of resistance development (9). Cellular ion homeostasis is an interesting target for antibiotic action, and the membrane potential is important for many essential processes of bacteria (30), e.g., ATP generation (31, 32), cell division (12), membrane transport (33), motility (34), and persister formation (35).

Dissecting the ion flux triggered by lugdunin, we noted that lugdunin treatment quickly led to an acidification of the *S. aureus* cytoplasm (Fig. 3), showing that protons are transported across the cytoplasmic membrane into the bacterial cytoplasm. The pH drop caused by lugdunin in bacterial cells is in accordance with the *in vitro* evidence we had previously gained for lugdunin-mediated proton transport across artificial lipid bilayers (11). We here investigated if lugdunin is a strict protonophore or if other ions can be transported as well. Among the set of reference ionophores we used for comparison in side-by-side experiments, CCCP is a protonophore, valinomycin is a selective potassium carrier ionophore, and gramicidin A forms an ion channel that is less selective and allows the passage of protons and other monovalent cations like Na^+^ and K^+^ across the cytoplasmic membrane (36–43). Our data indicate that lugdunin allows the passage of different monovalent cations across the cytoplasmic membrane of *S. aureus*, as its capacity to cause depolarization or hyperpolarization was strongly influenced by the ion species and concentrations in the surrounding medium. The effects of lugdunin were mostly comparable to gramicidin A, although lugdunin seems to possess a slight preference for K^+^ over Na^+^ (Fig. 4A), which gramicidin A did not show. The quick and prominent acidification of the *S. aureus* cytoplasm mediated by lugdunin further implies a high affinity for and capacity to transport protons. In clear contrast, CCCP was almost unaffected by the varying buffer conditions and always resulted in depolarization, while valinomycin only depolarized the membranes when the surrounding buffers contained K^+^, and caused a hyperpolarization in the absence of salts or when buffers contained only NaCl (Fig. 4). For valinomycin, it was previously observed that either depolarization or hyperpolarization occurs depending on the external and internal potassium concentrations (25, 44). Moreover, the ability of valinomycin to transport Na^+^ is about four orders of magnitude lower than that for K^+^ (25, 45). Our transport experiments using artificial lipid vesicles corroborated our data obtained for whole cells, demonstrating that not only protons but also K^+^ and Na^+^ are transported by lugdunin. The vesicle data showed a higher transport affinity for K^+^ over Na^+^ ions, confirming the trend observed in the membrane potential assays with *S. aureus*. Rapid proton translocation caused by lugdunin in lipid vesicles had previously been demonstrated (11). In addition, complexation of several monovalent cation species (i.e., K^+^, Na^+^, and Li^+^) by lugdunin occurred in the nonpolar environment of the picrate *in vitro* assay, with the order of selectivity being K^+^ > Na^+^ > Li^+^. Valinomycin also interacted with these three cations (K^+^ > Na^+^ > Li^+^) in our assay, and co-crystal structures of valinomycin with potassium picrate or sodium picrate have been described (19, 20). The considerable differences between those two valinomycin structures are probably due to the specific cavity size for ion binding that accommodates the atomic radius of K^+^ better than the smaller Na^+^, thereby reflecting the exceptional selectivity of valinomycin toward K^+^ over Na^+^ (45). Valinomycin can also form a weak complex with lithium (21).

The reference ionophore most strongly resembling lugdunin activity in all of our assays, gramicidin A, is a linear peptide consisting of 15 strictly altered L- and D-amino acids that form β-helices in lipid membranes building an ion channel consisting of the head-to-head dimer that allows permeation of protons and alkali cations (42, 43, 46–48). A biophysical/computational study by our co-authors on the interaction of lugdunin with membranes *in vitro* with the aim of determining if lugdunin serves as a mobile ion carrier or forms a multimeric channel across lipid membranes was published while our study was under review. The authors show that lugdunin forms water-filled channels in lipid membranes by building ring stacks that are connected via hydrogen bonds (24). Four molecules of the cycloheptapeptidic lugdunin assemble to build a dioleoylphosphocholine (DOPC) membrane-spanning channel. Those channel structures are reminiscent of the self-assembling nanotubular ion channels reported previously for even-numbered cyclopeptides with alternating D- and L-amino acid designs (49, 50). The lugdunin channel allows the permeation of monovalent cations, and conductance follows the lyotropic series (K^+^ > Na^+^ > Li^+^), which is a result of the increasing dehydration enthalpies of different cations and characteristic of membrane channels with a diffusive pore, like gramicidin A. Moreover, the effective pore size of the lugdunin channel was determined to be 3.66 Å by *in silico* analysis, which is close to the 4 Å pore diameter of gramicidin A (24). A single file of water molecules is expected in the lugdunin nanotube (24), similar to that described for gramicidin A (43, 51). As metal ions must be partially dehydrated to fit into the channel, the dehydration enthalpy is the decisive parameter that determines how well or whether an ion can pass (52). Of note, dehydration enthalpies are much larger for divalent cations due to their double positive charge (ΔH_K^+^_ ≈ 330 kJ mol^−1^ < ΔH_Na^+^_ ≈ 410 kJ mol^−1^ < ΔH_Li^+^_ ≈ 500 kJ mol^−1^ < ΔH_Ca^2+^_ ≈ 1,570 kJ mol^−1^ < ΔH_Mg^2+^_ ≈ 1,890 kJ mol^−1^ [53]), precluding their transport through the lugdunin and gramicidin A channels. These biophysical results are in full agreement with the live cell as well as *in vitro* results of our study, where we conclude that the ion selectivity of lugdunin closely matches that of gramicidin A and differs from that of the mobile ion carriers CCCP and valinomycin.

We had previously noted that lugdunin analogs, generated by acetylation, methylation, or ring expansion of the thiazolidine heterocycle, were inactive (11). Here, we found that the binding of K^+^, Na^+^, and Li^+^ by N-acetyl-lugdunin was reduced in the picrate assay compared to unmodified lugdunin. While the underlying reasons are yet unclear, hypotheses involve steric hindrance, altered basic properties of the thiazolidine nitrogen, and an altered system of hydrogen binding. Moreover, the new biophysical study shows that replacing thiazolidine with alanine resulted in a reduction of the partition coefficient by almost two orders of magnitude, highlighting the importance of the thiazolidine moiety for the insertion of lugdunin into membranes (24).

A synergism between lugdunin and dermcidin in the killing of *S. aureus* was noted before, which may play an important role in the natural microbiome–host context where both compounds are produced (10). To better understand this synergism from a mechanistic point of view, we combined a low, physiologically relevant dose of the dermcidin peptides DCD-1 or DCD-1L (5 µg/mL) with a concentration series of lugdunin. The concentration of DCD-1 in human sweat samples was previously found to be 1–10 µg/mL (54). In our assays, the dermcidin peptides alone showed no sign of membrane depolarization, in accordance with a previous study, where 50 µg/mL of DCD-1L only caused a slow loss of the *S. aureus* membrane potential, with no effect seen after 60 min and approx. 20% of depolarized cells after 90 min of treatment (55). We could also exclude a general loss of membrane integrity upon treatment with 5 µg/mL of DCD-1 alone and in combination with 4× MIC lugdunin (Fig. S7), in agreement with a previous report describing the absence of pores at 50 µg/mL of DCD-1L alone (56). It has been suggested that DCD-1 and DCD-1L form Zn^2+^-dependent oligomeric complexes in the bacterial membrane, thereby leading to ion channel formation (56, 57). Our data suggest that lugdunin and dermcidin-derived peptides act synergistically by strongly potentiating the depolarization of the bacterial cytoplasmic membrane, thereby leading to severe energy depletion in the cells even at compound concentrations substantially below the MIC of each individual agent. Our finding that lugdunin and its enantiomer are similar in their concerted action with DCD-1 implies the lack of direct molecular interaction between lugdunin and the dermcidin-derived peptides, which would be expected to require either one or the other structural configuration of lugdunin. Combinations of compounds that block the proton motive force (PMF) in *S. aureus* had previously been identified to act in a highly synergistic manner, when both the electrical and chemical components of the PMF, ∆ψ and ∆pH, were dissipated (58). By acting as an ionophore, lugdunin can benefit from the synergistic activity of several host- and commensal-derived membrane-active agents produced in its natural environment (10), thereby lowering its minimal antibacterial concentrations.

Lugdunin affected distinct eukaryotic cell lines to a very different extent. The epithelial cell lines A549 and HEK293T were much less susceptible than the monocytic suspension cell line THP-1. Moreover, when A549 and HEK293T were adherent before treatment, lugdunin showed almost no effect on their metabolic activity up to a concentration of 50 µg/mL. Also, HeLa cells were markedly less susceptible to lugdunin when they had adhered before treatment. Similarly, in a previous study, lugdunin was not toxic to primary human keratinocytes and primary human nasal cells up to the highest tested concentration of 10 µM (10). With regard to the mode of action of lugdunin in eukaryotic cells, we observed that mitochondria depolarized in the more sensitive HeLa cells after 15 min of compound exposure, suggesting a primary effect of lugdunin. This observation indicates that lugdunin can pass the plasma membrane of certain eukaryotic cells and the outer mitochondrial membrane to reach the energized inner mitochondrial membrane. Passage of the outer and depolarization of the inner mitochondrial membrane were also shown for hydrophobic valinomycin (>1,000 Da) (59) and even the large gramicidin A, the monomer composed of 15 amino acids (60). Although ionophore activities and corresponding ion imbalances at the site of the eukaryotic plasma membrane have not been investigated here, it is noteworthy that lugdunin was not hemolytic up to the highest tested concentrations of 50 µg/mL in experiments with primary human erythrocytes in previous studies (9), in contrast to the hemolytic ionophore gramicidin A (61). Of importance may be differences in the permeation of lugdunin into the lipid bilayer of the plasma membrane depending on the lipid composition. The new biophysical study showed that the partitioning of lugdunin into cholesterol-containing membranes is impeded hindering deep penetration into the membrane core (24). Notably, it has been shown that trypsin, which is used in cell culture for the detachment of adherent cells during splitting for cell passaging, promotes a release of cholesterol (62). A potentially lower amount of cholesterol directly after trypsinization may allow increased lugdunin partitioning into plasma membranes and diffusion to the mitochondria. In summary, antibacterial lugdunin concentrations are substantially lower than inhibitory concentrations against adherent (this work) or confluent (10) epithelial cells and even reduced further by synergistic interaction with host-derived antimicrobial peptides. Also, in mouse skin infection models (9, 10), lugdunin was well tolerated in a topic application.

### Conclusion

Compounds with ionophore activity are attractive as well as challenging molecules in antibiotic drug development. In recent years, some of them are also being evaluated and improved for killing cancer stem cells and multi-drug-resistant cancer cells (63, 64). Produced by a commensal of the human nasal microbiome, lugdunin has already proven efficacy *in vivo* by eradicating pathogens such as *S. aureus* from the nose and the skin. Our current study shows that lugdunin acts as a cation ionophore, depleting the energy resources of Gram-positive bacterial cells. The passage of different monovalent cations (e.g., H^+^, Na^+^, and K^+^) across the cytoplasmic membrane is promoted, and synergistic activity occurs with host peptides increasing membrane permeability for ions such as dermcidin-derived peptides present on the human skin. Mechanistically, the synergism arises by the amplification of membrane potential dissipation. As a microbiome-derived antibacterial compound, lugdunin has evolved for bacterial eradication on human epithelia, suggesting that it is optimized for both tolerance and efficacy at its physiological site of action. Its mode of action as a cation ionophore that rapidly depletes bacterial energy resources and the lack of a protein target reduce the risk of resistance development.

## MATERIALS AND METHODS

### Determination of antibacterial activity

The MIC was determined by the broth microdilution method according to the Clinical and Laboratory Standards Institute guideline using the direct colony suspension method with an inoculum of 5 × 10^5^ CFU/mL (65). Cells were incubated in cation-adjusted Mueller–Hinton broth (MHB), and MICs were read after 18–20 hours of incubation at 37°C.

### Membrane potential assay

Bacterial strains were grown to the exponential phase in lysogeny broth (LB) + 0.1% glucose, harvested, and resuspended to an optical density at 600 nm (OD_600_) of 0.5 in different buffers as indicated. Cell suspensions were incubated with 30 µM DiOC_2_(3) (Molecular Probes) for 15 min in the dark and transferred to a black flat-bottom 96-well microplate. Fluorescence was measured at an excitation wavelength of 485 nm and two emission wavelengths, 530 nm (green) and 630 nm (red), using a microplate reader (Tecan Infinite M200 pro or Tecan Spark). After initial fluorescence measurements to determine the membrane potential in the untreated state, cells were treated with lugdunin and control antibiotics at indicated concentrations. Green/red fluorescence measurements were continued over a time period of 15 min. In assays with dermcidin-derived peptides, additional measurements after 30 and 60 min were included. For membrane potential measurements in *E. coli*, 15 µg/mL PMBN was included in the buffer, and the time period for fluorescence measurements after compound treatment was extended to 240 min. Experiments were performed as a set of minimum of three biological replicates with two technical replicates each.

### Assessment of intracellular pH

Changes in bacterial intracellular pH were measured using the pH-sensitive dye BCECF-AM. *S. aureus* NCTC8325 was grown to the exponential phase in LB + 0.1% glucose, harvested, washed two times with PBS, and concentrated fourfold by resuspending in PBS. One milliliter of loading buffer consisting of 40 µL PowerLoad Concentrate (100×) (Invitrogen, Thermo Fisher Scientific), 20 µL BCECF-AM (2.5 µg/µL), 40 µL Probenecid (100×; 77 mg/mL), and 900 µL PBS were added to 1 mL of the concentrated cells and incubated for 30 min at 30°C, protected from light. The cell suspension was centrifuged, washed 2× with PBS + 5 mM glucose, resuspended in 4 mL of PBS containing 25 mM glucose, and further incubated for 5 min at 37°C in the dark. One hundred microliters of the cell suspension per well was added to a black flat-bottom 96-well microplate, and fluorescence (Ex1: 490 nm and Em1: 530 nm; Ex2 440 nm and Em2: 530 nm) was measured every 30 s for 5 min in a microplate reader (Tecan Spark) to determine the intracellular pH in the untreated state. The program was paused, antibiotic compounds were added at the indicated concentrations, and fluorescence was recorded every 30 s for 10 min. Finally, 20 µM of nigericin was added to each sample, and fluorescence was measured for an additional 5 min. For each sample, the fluorescence ratio of 490 nm/440 nm was determined.

In order to correlate the fluorescence ratios (490/440 nm) to the intracellular pH, a calibration curve with potassium phosphate buffers (135 mM KH_2_PO_4_/20 mM NaOH and 110 mM K_2_HPO_4_/20 mM NaOH) with six different pH values in the range of pH 6.5 to pH 8 was prepared according to Clementi et al. (66). The intracellular pH was interpolated for each sample from the calibration curve.

### Microscopy

To monitor the delocalization of cell division proteins, *B. subtilis* YK020 (expressing YFP-FtsA) (67) and *B. subtilis* 1981 (expressing GFP-MinD) (12) were induced with 1 mM isopropyl-β-D-thiogalactopyranosid (IPTG) or 0.1% xylose, respectively, and grown to an OD_600_ of 0.15–0.25 in LB. Subsequently, strains were treated for 20 min with different antimicrobial compounds as indicated and analyzed by fluorescence microscopy. To detect the formation of large pores, co-staining with SYTO 9 and PI was performed as described previously (11). *B. subtilis 168* trpC2 was grown in LB to the mid-exponential phase and treated for 10 min with different compounds as indicated. Cells were stained using a mixture of SYTO 9 (6 µM) and PI (30 µM) for 10–15 min and subsequently analyzed by fluorescence microscopy. The integrity of the peptidoglycan sacculus was monitored by the formation of blebs following an acetic acid/methanol fixation method as described previously (68). Briefly, *B. subtilis* 168 trpC2 was grown in MHB to the mid-exponential phase, then treated for 30 min with different antibiotics as indicated. Samples were fixed with 1:3 (vol:vol) mixture of acetic acid and methanol and visualized by phase contrast microscopy.

In all microscopy experiments, bacterial cells were immobilized on 1% agarose-covered slides and immediately analyzed via microscopy. Brightfield, phase contrast, and fluorescence microscopy were performed using a Zeiss Axio Observer Z1 automated microscope equipped with an alpha Plan-Apochromat 100×/1.46 oil Ph3 objective, a C Plan-Apo 63×/1.4 oil DIC objective (Zeiss), and an Orca Flash 4.0 V2 camera (Hamamatsu), or a Zeiss Axio Scope.A1 equipped with an EC Plan-Neofluar 100×/1.30 oil objective (Zeiss) and an AxioCam ICm1 Rev.1 (Zeiss). Images were processed using the ZEN 2.3 image analysis software package (Zeiss).

For microscopy of eukaryotic cell lines, cells were seeded in a microscopy chamber slide (cat no. 543079, Greiner, Germany) at a cell number of 2 × 10^4^ cells per well and incubated overnight at 37°C, 5% CO_2_, 95% relative humidity to allow adherence of the cells. On the next day, a fresh cell culture medium (Dulbecco’s modified Eagle medium [DMEM] for A549, RPMI for HeLa) containing the fluorescent dyes JC-1 (5,5,6,6-tetrachloro-1,1,3,3- tetraethylbenzimidazolylcarbocyanine iodide) (5 µg/mL) and 4′,6-diamidino-2-phenylindole (DAPI; 1 µg/mL) was added, and cells were incubated at 37°C, 5% CO_2_, 95% relative humidity for 15 min. The medium was exchanged, and compounds were added at the indicated concentrations. After 10–20 min of incubation, cells were analyzed via fluorescence microscopy. Micrographs were obtained using a Nikon Eclipse Ti automated microscope equipped with a Perfect Focus system (Nikon Instruments Europe BV, Netherlands), CFI Plan-Apo DM 100×/1.45 oil Ph3 objective (Nikon), and an Orca Flash 4.0 camera (Hamamatsu, Photonics, Japan). Image acquisition and analysis were performed via the NIS elements AR software package (Nikon). Images were processed using ImageJ (Fiji) (69).

### Chemical synthesis of lugdunin

The fibupeptide of interest, lugdunin, its enantiomer, and the N-acetylated lugdunin have been prepared as previously described (9, 11). Structures are depicted in Fig. S1. Additionally, the natural lugdunin from *S. lugdunensis* cultures (9) served as an antibacterial agent and showed identical properties as the synthetic lugdunin, both in chemical analysis and in the here-described biological assays.

### Picrate assay

The picrate assay was conducted according to Audhya und Russell (17) and Bourgoin et al. (18). Picric acid aqueous solution (0.175 M, 160 mg picric acid, 0,70 mmol, in 4 mL H_2_O) was combined with 10 mL of a 1 M sodium hydroxide solution (0.1 M) and diluted with 36-mL deionized water to afford the alkaline picrate solution (Na^+^C_6_H_2_N_3_O_7_^−^). The same process is applied to obtain a potassium picrate solution (K^+^C_6_H_2_N_3_O_7_^−^) and a lithium picrate solution (Li^+^C_6_H_2_N_3_O_7_^−^). The compounds of interest (0.029 µmol) lugdunin, erythromycin, and daptomycin, each, were dissolved in 1 mL dichloromethane (p.a.) in a 5 mL glass vial with a tight seal. 0.5 mL of either lithium, sodium, or potassium picrate solution was added, and the solution was emulsified by vigorous shaking for 10 min. The aqueous upper layer was discarded, and the organic dichloromethane phase (0.3 mL) was transferred into a quartz cuvette for UV-Vis (ultraviolet-visible light) absorbance in a spectroscopy instrument (Varian Cary 50 Scan). The UV absorbance was measured at 370 nm.

### Ion transport in artificial lipid membrane vesicles

#### Preparation of large unilamellar vesicles

To analyze the transport capability of lugdunin dependent on the presence of different monovalent metal ion concentrations, LUVs composed of POPC (Avanti Polar Lipids, Alabaster, US) filled with the pH-sensitive fluorescent dye pyranine (8-hydroxypyrene-1,3,6-trisulfonic acid) were employed. A lipid film was prepared by drying 2 mg POPC from a chloroform stock solution first under a gentle nitrogen stream and then for 3 hours *in vacuo*. The film was rehydrated with 1 mL 10 mM HEPES and 0.5 mM pyranine doped with 0–500 mM MCl (pH 7.4, M = K, Na, or Li) and was incubated for 30 min. Vortexing three times in 5-min intervals yielded a suspension of multi-lamellar vesicles transformed into LUVs by extrusion for 31 times through a polycarbonate membrane with a nominal pore diameter of 200 nm (Avestin, Ottawa, Canada). Extravesicular pyranine molecules were removed via size exclusion chromatography (Illustra NAP 25 G25 column, GE Healthcare). The final phospholipid concentration was determined by quantifying the phosphate content as described previously (11).

#### Proton translocation assay

Fluorescence spectroscopic experiments were conducted with a FP-6500 spectrofluorometer (Jasco Deutschland GmbH, Pfungstadt, Germany) at 20°C using a 10 mm × 4 mm quartz cuvette (Hellma, Müllheim, Germany) under constant stirring. LUVs filled with pyranine were diluted in the same buffer used for vesicle preparation (containing 0–500 mM MCl, M = K, Na, or Li) with pH 6.4 to a final phospholipid concentration of 50 µM and a volume of 792 µL. Fluorescence intensity was recorded over time with λ_ex_ = 458 nm, λ_em_ = 512 nm and band widths of 3 nm. After acquiring a baseline for 100 s, 8 µL of 20 µM lugdunin in isopropanol was added (final concentration 0.2 µM, peptide-to-lipid ratio 1:250), and changes in fluorescence intensity were monitored for 500 s. Proton influx would lead to the protonation of pyranine molecules and, thus, fluorescence quenching. To lyse the vesicles and equilibrate the pH gradient, 13 µL of 3% (wt/vol) lauryldimethylamine-N-oxide (LDAO) was added.

#### Data analysis

All data points were normalized to the fluorescent intensity before the addition of lugdunin and after vesicle lysis. The initial linear slope of the time traces was extracted as a measure of proton transport activity and corrected for the baseline drift. The slope obtained in the absence of any metal cation was subtracted from all other data points.

### Determination of activity on eukaryotic cells

The activity of lugdunin, reference ionophores, and cycloheximide against different human cell lines was determined using the 7-hydroxy-3H-phenoxazin-3-one-10-oxide (resazurin) assay. A549 human epithelial cells from lung carcinoma and HEK293T human embryonic kidney cells were cultivated in DMEM, and the human cervical carcinoma epithelial cell line HeLa and human leukemia monocytic cell line THP-1 were cultivated in RPMI. Both cell culture media were supplemented with 10% fetal bovine serum.

For direct treatment, a twofold serial dilution of the test compounds was prepared in a microplate and seeded with trypsinized A549 or HEK293T cells, or THP-1 suspension cells to a final cell concentration of 1 × 10^4^ cells per well. To determine the activity of the agents against cells that had previously been allowed to settle and adhere to the surface, cells were seeded to a final cell concentration of 1 × 10^4^ cells per well and incubated overnight (~18 hours) at 37°C, 5% CO_2_, 95% relative humidity, followed by treatment with the different compounds in fresh cell culture medium on the next day.

For both conditions, cells were incubated for 24 hours in the presence of the test compounds at 37°C, 5% CO_2_, 95% relative humidity, followed by the addition of resazurin at a final concentration of 200 µM and subsequent incubation overnight. Cell viability/metabolic capacity was assessed by determining the reduction of resazurin to the fluorescent resorufin. Fluorescence was measured in a microplate reader (Tecan Infinite M200) at an excitation wavelength of 560 nm and an emission wavelength of 600 nm, and data were evaluated in relation to the untreated control.

### Bioreporter assays

For the analysis of cellular stress responses upon antibiotic treatment, bacterial reporter strains were used carrying the promoter of the *yorB*, *bmrC*, *helD*, *ypuA,* or *liaI* genes fused to either the firefly luciferase (liquid assay) or the beta-galactosidase reporter gene (agar-based assay) in strain *B. subtilis* 1S34 (70), a sporulation-deficient derivative of *B. subtilis* 168 *trpC2*.

The promoter assay in liquid media was carried out as previously described (14, 71). Briefly, reporter strains were cultured overnight in LB with 5 µg/mL erythromycin at 37°C and 190 rpm. Cultures were diluted to an OD_600_ of 0.05 in either Belitzky minimal medium (BMM) (72) (*bmrC* strain) or LB (all other strains) with 5 µg/mL erythromycin, grown to an OD_600_ of 0.4 (*bmrC* strain) or 0.9 (all other strains) at 37°C and 190 rpm, and subsequently diluted to an OD_600_ of 0.02. Serial twofold dilutions of the different antibiotics in LB or BMM were prepared in white 96-well flat-bottom polystyrene microplates and were inoculated with the respective diluted bacterial cell suspension. Microplates were incubated at 37°C without shaking for a predetermined time period depending on the induction kinetics of the reporter strain: 1 hour for the *liaI* and *ypuA* strains, 1.5 hours for the *helD* strain, 3.5 hours for the *yorB* strain, and 4 hours for the *bmrC* strain. After incubation in the presence of antibiotics, 0.1 M citrate buffer (pH 5) containing 2 mM luciferin was added, and flash luminescence was measured using a microplate reader (Infinite M200 PRO, Tecan).

Agar-based bioreporter assays were performed according to Wex et al. (13). Briefly, inoculated from overnight cultures, the different *B. subtilis* bioreporter strains were grown in LB with 100 µg/mL spectinomycin to an OD_600_ of 1. Cells were concentrated 1:10 (4,700 rpm, 4°C) and used to inoculate the soft agar. LB soft agar was used for the *ypuA*, *liaI*, *yorB,* and *helD* bioreporter constructs, and Belitzky minimal soft agar was used for the *bmrC* construct. Cell numbers were adjusted to 3 × 10^7^ CFU/mL, except for the *ypuA* strain (6 × 10^7^ CFU/mL). The soft agar was supplemented with 5 mM MgCl_2_ and 150 µg/mL X-Gal. For the testing of antibiotic compounds, the soft agar containing the respective reporter strain was poured into petri dishes and allowed to solidify for approximately 15 min. Subsequently, compounds were spotted on the agar, and the plates were incubated overnight at 30°C for LB and 37°C for Belitzky minimal soft agar, respectively.

### *In vitro* transcription/translation assay

The *in vitro* transcription/translation assay was performed as described by Hörömpöli et al*.* (16). The assay is based on an *E. coli* S30 extract (73) and uses the pBESTluc plasmid (Promega Corporation, Madison, WI, USA) encoding the firefly luciferase reporter gene under the control of a tac promoter, allowing the detection of the inhibition of transcription or translation processes by measuring luminescence. The assay reaction mixture also contained the following supplements: 2.5 mM ATP, 0.5 mM GTP, 0.5 mM UTP, 0.5 mM CTP, 20 amino acids (0.04 mM each), an ATP-regenerating system (creatine phosphokinase/phosphocreatine), 3.2% (wt/vol) polyethylene glycol 600, 8 mM putrescine, and 2 mM dithiothreitol in an appropriate buffer system (40 mM triethylamine [pH 7.5], 140 mM potassium acetate, 8 mM magnesium acetate, 20 mM ammonium acetate, and 1.4 mM spermidine). *In vitro* coupled transcription/translation reactions in the presence of a concentration series of lugdunin were performed for 2 hours at 25°C. The translation inhibitor pactamycin was used as a control. After the addition of the substrate luciferin, chemiluminescence was recorded in a microplate reader (Infinite M200, Tecan).

## SUPPLEMENTAL MATERIAL

Supplemental Figures and Tables mbio.00578-24-s0001.pdf Figures S1-S7 and Tables S1 and S2.

ASM does not own the copyrights to Supplemental Material that may be linked to, or accessed through, an article. The authors have granted ASM a non-exclusive, world-wide license to publish the Supplemental Material files. Please contact the corresponding author directly for reuse.
