# Supplementary material for: The microbiome-derived antibacterial lugdunin acts as a cation ionophore in synergy with host peptides
Source: mBio. 2024 Aug 12;15(9):e00578-24. doi: 10.1128/mbio.00578-24 (PMC11389392; doi:10.1128/mbio.00578-24)
Supplement: Supplemental Figures and Tables — Figures S1-S7 and Tables S1 and S2. [file mbio.00578-24-s0001.pdf]

Supplemental Figures and Tables

The microbiome-derived antibacterial lugdunin acts as a cation ionophore in synergy with host peptides

Anne Berscheid[a,f#], Jan Straetener[a], Nadine A. Schilling[b], Dominik Ruppelt[c], Martin C. Konnerth[b], Birgit Schittek[d], Bernhard Krismer[e,f,g], Andreas Peschel[e,f,g], Claudia Steinem[c,h] , Stephanie Grond[b,g], Heike Brötz-Oesterhelt[a,f,g#]

[a] Interfaculty Institute of Microbiology and Infection Medicine, Microbial Bioactive Compounds, University of Tübingen, 72076 Tübingen, Germany

[b] Institute of Organic Chemistry, University of Tübingen, 72076 Tübingen, Germany

[c] Georg-August-Universität Göttingen, Institute of Organic and Biomolecular Chemistry, 37077 Göttingen, Germany

[d] Department of Dermatology, Division of Dermatooncology, University of Tübingen, 72076 Tübingen, Germany

[e] Interfaculty Institute of Microbiology and Infection Medicine, Infection Biology, University of Tübingen, 72076 Tübingen, Germany

[f] German Centre for Infection Research (DZIF), Partner Site Tübingen, 72076 Tübingen, Germany

[g] Cluster of Excellence EXC 2124 - Controlling Microbes to Fight Infections, 72076 Tübingen, Germany

[h] Max-Planck-Institute for Dynamics and Self Organization, 37077 Göttingen, Germany

[#] Address correspondence to Heike Brötz-Oesterhelt, heike.broetz-oesterhelt@uni-tuebingen.de or Anne Berscheid, anne.berscheid@uni-tuebingen.de

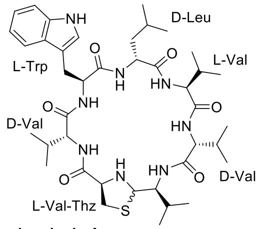

Lugdunin A
Chemical Formula: $C_{40}H_{62}N_8O_6S$
MW: 783.0460

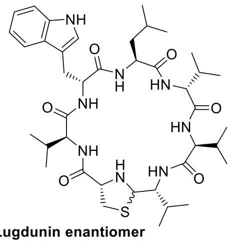

Lugdunin enantiomer
Chemical Formula: $C_{40}H_{62}N_8O_6S$
MW: 783.0460

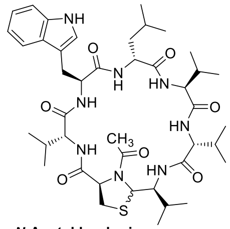

***N*-Acetyl lugdunin**
Chemical Formula: $C_{42}H_{64}N_8O_7S$
MW: 825.0830

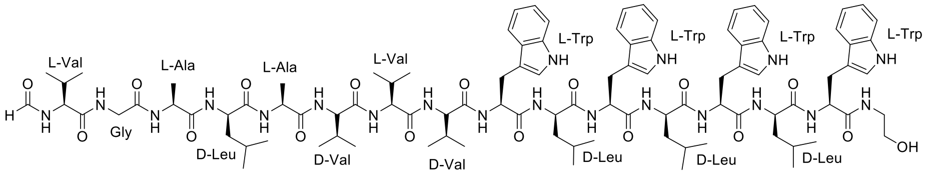

Gramicidin A1
Chemical Formula: $C_{99}H_{140}N_{20}O_{17}$
MW: 1882.3320

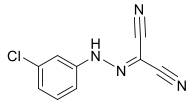

CCCP
Carbonyl cyanide *m*-chlorophenylhydrazone
Chemical Formula: $C_9H_5ClN_4$
MW: 204.6170

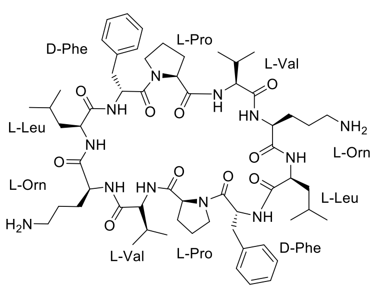

Gramicidin S
Chemical Formula: $C_{60}H_{92}N_{12}O_{10}$
MW: 1141.4700

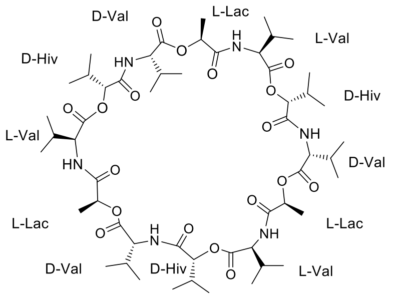

Valinomycin
Chemical Formula: $C_{54}H_{90}N_6O_{18}$
MW: 1111.3380

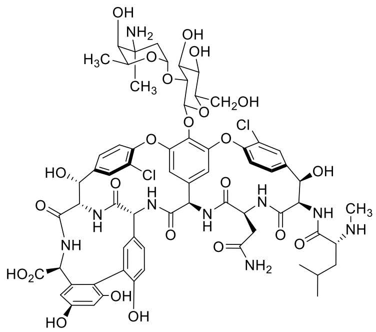

Vancomycin
Chemical Formula: $C_{66}H_{75}Cl_2N_9O_{24}$
MW: 1449.2650

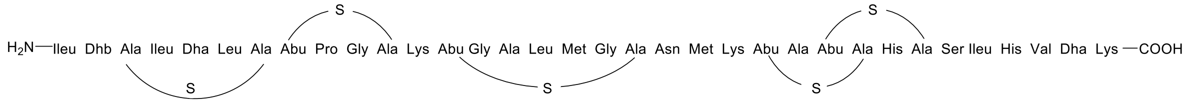

Nisin
Chemical Formula: $C_{143}H_{230}N_{42}O_{37}S_7$
MW: 3354.07

SSLLEKGLDGAK KAVGGLGKLGKD AVEDLESVGKGA VHDVKDVLDSV

DCD-1 (47-mer), a dermcidin derived truncated peptide
Chemical Formula: $C_{204}H_{348}N_{56}O_{70}$
MW: 4702

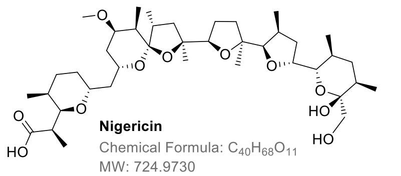

Nigericin
Chemical Formula: $C_{40}H_{68}O_{11}$
MW: 724.9730

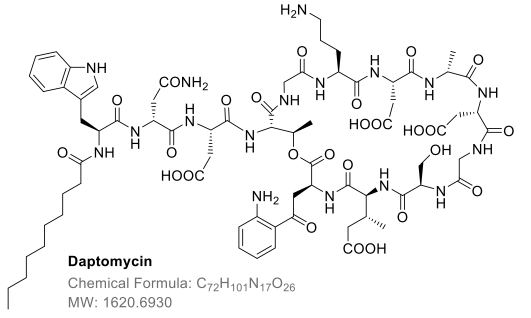

Daptomycin
Chemical Formula: $C_{72}H_{101}N_{17}O_{26}$
MW: 1620.6930

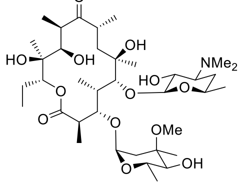

Erythromycin
Chemical Formula: $C_{37}H_{67}NO_{13}$
MW: 733.9370

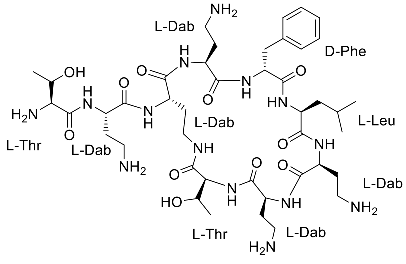

Polymyxin B nonapeptide (PMBN)
Chemical Formula: $C_{43}H_{74}N_{14}O_{11}$
MW: 963.1520

Figure S1. Structures of antibacterial agents used in this study.

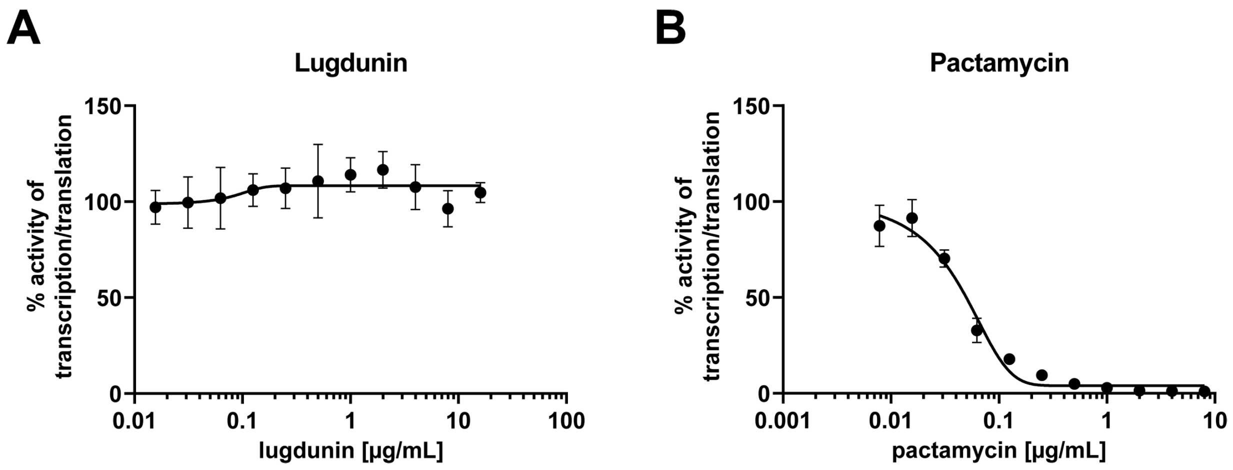

Figure S2. *In vitro* coupled transcription and translation assay. Lugdunin did not interfere with the transcription or translation of the firefly luciferase reporter in a cell-free assay (A). The translation inhibitor pactamycin was used as a positive control (B). The assay was performed with 3 replicates, error bars show the standard deviation (SD).

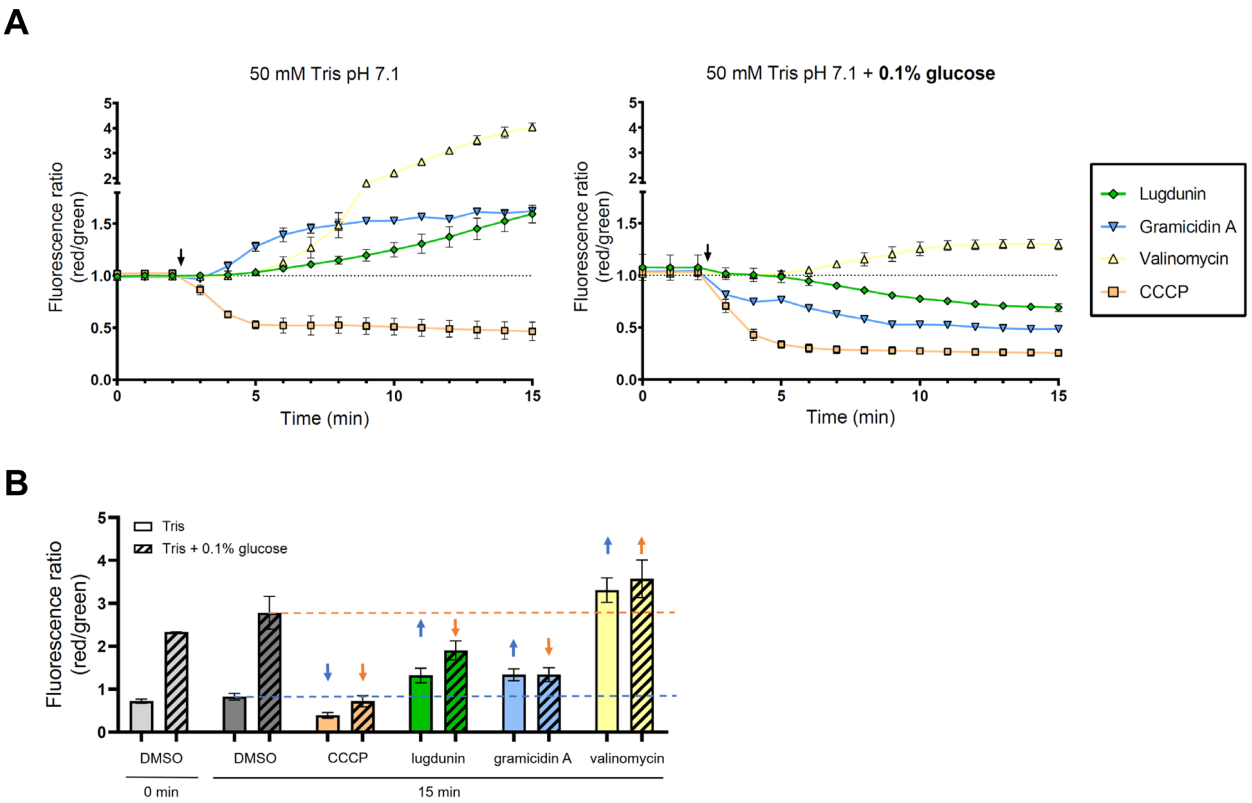

Figure S3. Membrane potential assay with *S. aureus* NCTC8325 in the absence and in the presence of glucose. (A) In Tris buffer without additional salts, lugdunin (3 µg/mL, 3.83 µM, 1x MIC) and gramicidin A (5 µM, 0.5x MIC) switched from hyperpolarization in the absence of glucose (left graph) to depolarization when 0.1% glucose was present (right graph). Valinomycin (5 µM, 0.25x MIC) hyperpolarized and CCCP (5 µM, 0.5x MIC) depolarized the cytoplasmic membranes under both conditions. The time point of compound addition is indicated by a black arrow. All data of the lugdunin, gramicidin A, valinomycin and CCCP treated samples were normalized to the untreated DMSO control, which was set to a fluorescence ratio (red/green) of 1 for every time point. The experiment was performed with 2 biological replicates with 2 technical replicates each, error bars show the SD. **(B)** Membrane energization of *S. aureus* NCTC8325 in Tris with and without glucose. When glucose is present in the Tris buffer (striped bars), the cytoplasmic membrane is more strongly polarized as indicated by the higher red/green fluorescence ratio compared to Tris buffer without glucose (unstructured bars). As

transmembrane ion gradients strive to equalize in the presence of ionophores (their passage limited by ionophore selectivities), compound treatment results either in depolarization (down arrows) or hyperpolarization (up arrows) depending on the initial polarization level in untreated control (DMSO) samples as well as the ion preference of the specific ionophore. The dashed blue and orange lines mark the polarization level of the DMSO controls in Tris buffer without and with glucose, respectively, and the color of the arrows refers to those two baselines. Ionophore exposure strongly reduces the differences between the initial membrane polarization levels (+/-glucose) towards an ionophore specific endpoint of the membrane potential. Data correspond to 0 min and 15 min values of the experiments presented in Figure S3A without normalization to the untreated DMSO control.

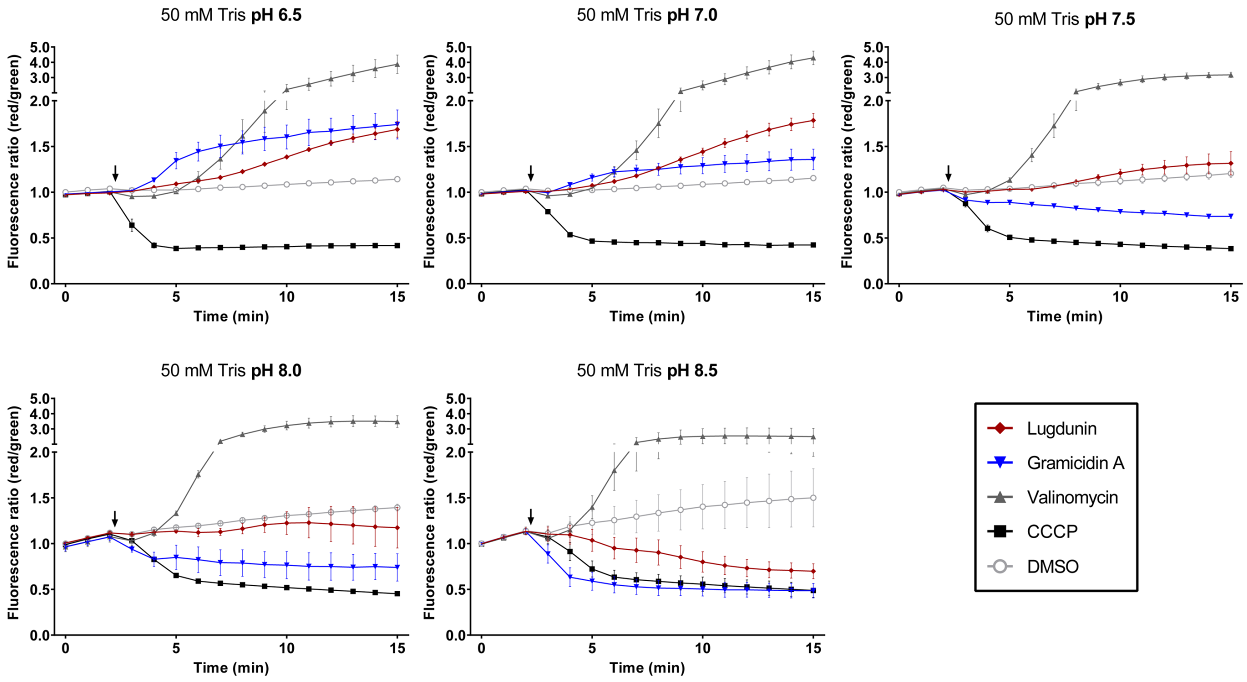

Figure S4. Membrane potential assay with *S. aureus* NCTC8325 in buffer with different pH. The experiment was conducted in 50 mM Tris buffer adjusted to pH 6.5, pH 7.0, pH 7.5, pH 8.0 or pH 8.5 without any additional salts. Lugdunin (3 µg/mL, 3.83 µM, 1x MIC) and gramicidin A (5 µM, 0.5x MIC) switched from hyperpolarization at lower pH to depolarization at more alkaline pH when compared to the untreated DMSO control. Valinomycin (5 µM, 0.25x MIC) hyperpolarized and CCCP (5 µM, 0.5x MIC) depolarized the cells in the complete pH range from 6.5 to 8.5. The time point of compound addition is indicated by a black arrow. The experiment was performed with minimum 2 biological replicates with 2 technical replicates each, error bars show the standard error of the mean (SEM). All data of the lugdunin, gramicidin A, valinomycin and CCCP treated samples were normalized to the untreated DMSO control. The 0 min value of the DMSO control was set to a fluorescence ratio (red/green) of 1 for each experiment.

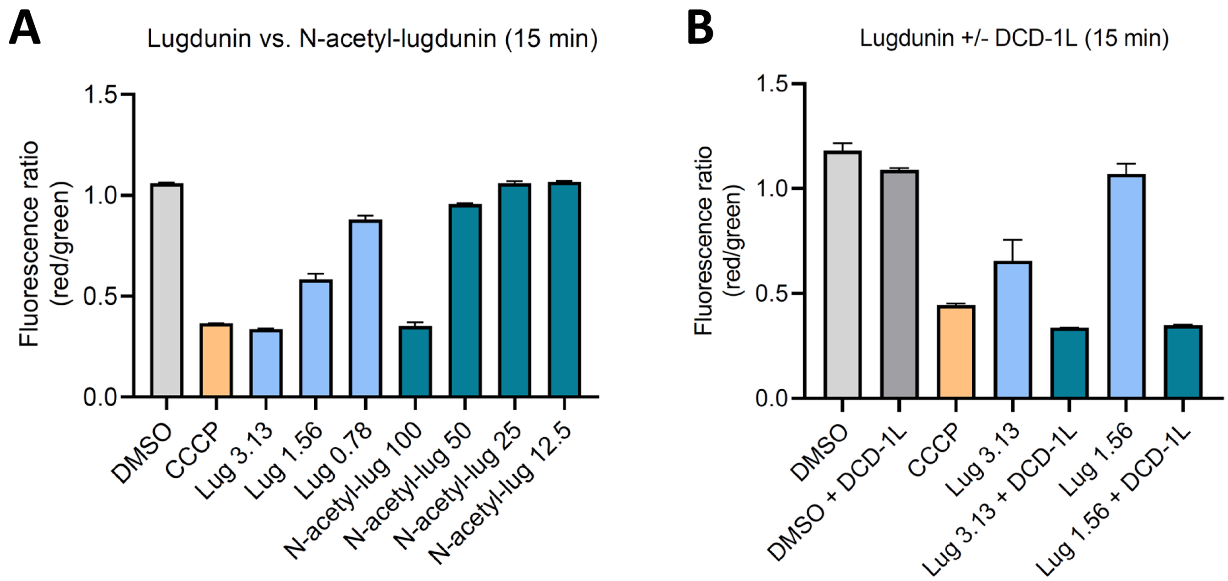

Figure S5. Membrane potential assay with *S. aureus* NCTC8325 to analyze the activity of N-acetyl-lugdunin (A) and dermcidin derived peptide DCD-1L (B). (A) The derivative N-acetyl lugdunin (Figure S1) showed membrane depolarization only at very high compound concentrations (100 µg/mL), in accordance with its high MIC (≥100 µg/mL). **(B)** DCD-1L (5 µg/mL), when applied in combination with lugdunin, strongly enhanced *S. aureus* membrane depolarization, as observed for DCD-1 (compare Figure 6). CCCP (5 µM) was used as positive control for membrane depolarization in all assays. The assays were conducted in 50 mM Tris buffer with 100 mM KCl **(A)** or PBS **(B)**, respectively, with 2 replicates. Data represent the mean of the recorded fluorescence ratio (red/green) after 15 min of treatment with the respective compounds. Error bars show the SD.

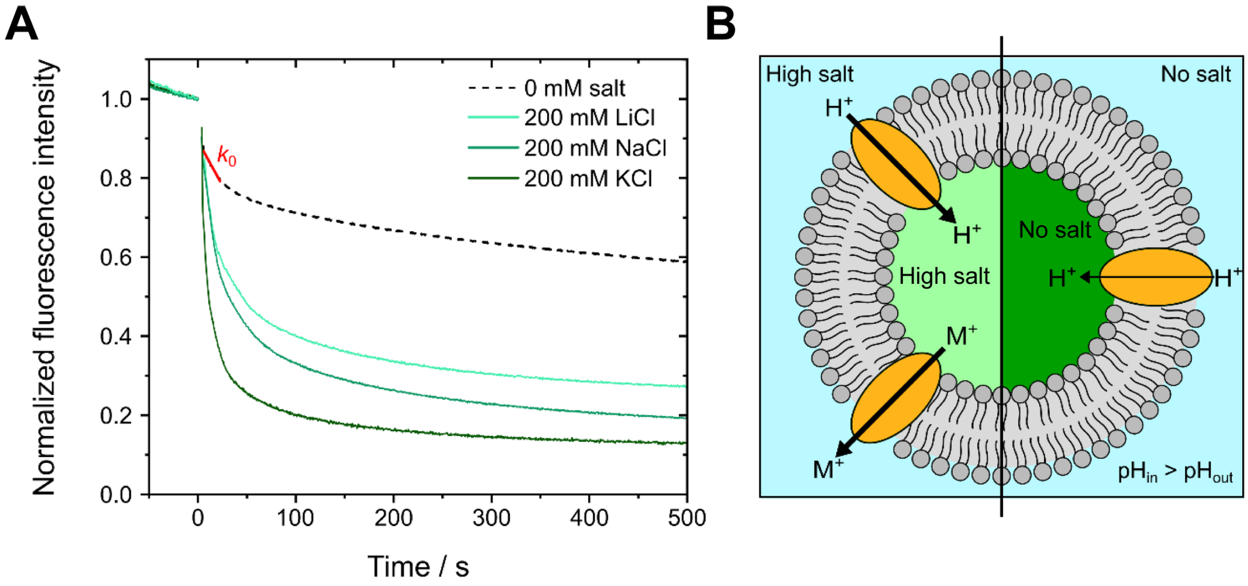

E1	$k_0 = \frac{k_{max}\, c(M^+)}{K_T + c(M^+)}$

Figure S6. Lugdunin-mediated ion transport in artificial membrane vesicles. The proton transport rates k_0 dependent on the concentration of the metal cation $c(M^+)$ follow a Michaelis-Menten-like kinetic. With the assumption that no transmembrane potential is generated as a result of a 1:1 antiport of protons and metal cations, the formal consideration of a Michaelis-Menten-like kinetic (1) leads to equation E1, with k_{max} denoting the maximum transport rate and K_T the transport constant. Similar to the Michaelis-Menten constant K_M, K_T corresponds to the metal ion concentration at the half maximum transport rate and is a measure of the transport affinity of lugdunin for the corresponding cation. **(A)** Exemplary proton transport curves obtained after adding lugdunin to vesicles filled with the pH-sensitive dye pyranine and exposed to a pH gradient (pH_{in} = 7.4, pH_{out} = 6.4) in the presence of no salt, 200 mM KCl, 200 mM NaCl or 200 mM LiCl. A peptide-to-lipid ratio of 1:250 was used. Proton influx leads to fluorescence quenching. Equation E1 was used to determine the transport constants K_T for the different ions with k_{max} the maximum transport rate and $c(M^+)$ the metal ion concentration. **(B)** The proton transport induced by lugdunin (symbolized by yellow ovals) across the membrane of pyranine-filled large unilamellar vesicles

(LUVs) was monitored in the absence and presence of different metal ion concentrations (M^+ = K^+, Na^+ or Li^+). In case of a high metal ion concentrations inside and outside of the vesicle (left), charge equilibration was achieved by the efflux of metal ions enabling a strong proton influx and quenching of pyranine, which resulted in a decreased fluorescence intensity. In the absence of metal ions (right), the proton transport was inhibited by the buildup of positive charge inside the vesicle (membrane potential) hampering further influx of H^+. As a consequence, less pyranine molecules are quenched and the observed decrease in fluorescence intensity is smaller.

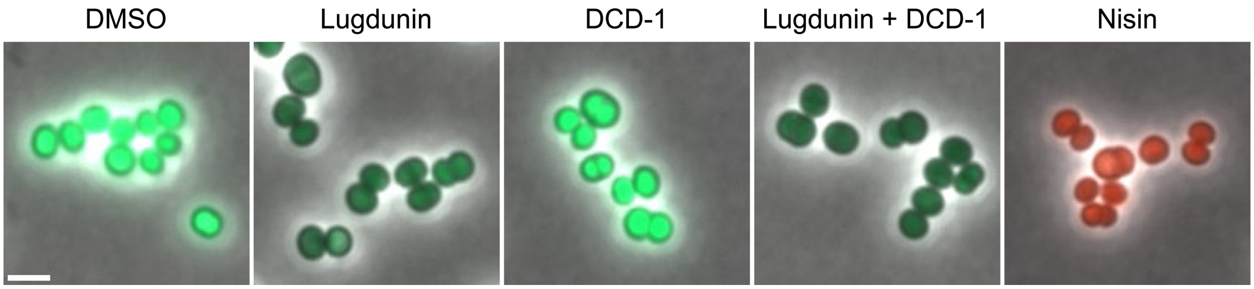

Figure S7. Analysis of cytoplasmic membrane integrity in *S. aureus* NCTC8325. Cells were co-stained with a mixture of SYTO 9 and propidium iodide (PI) and incubated with the respective compounds for 20 min. Treatment with lugdunin (12.5 µg/mL, 4x MIC), DCD-1 (5 µg/mL) or both compounds at the same time (12.5 µg/mL lug + 5 µg/mL DCD-1) did not lead to the formation of large pores in *S. aureus*. The positive control nisin (100 µg/mL, crude nisin preparation) mediated pore formation allowing the entry of the membrane-impermeant DNA dye PI (red). Of note, cells with depolarized membranes appear less bright green after staining with SYTO 9 (2). Scale bar 2 µm.

Table S1. Antimicrobial activity of lugdunin against different *E. coli* efflux mutants in the absence and presence of polymyxin B nonapeptide (PMBN).

	**Lugdunin MIC [µg/mL]**	
**Strain**	w/o PMBN	+ 15 µg/mL PMBN
E. coli ATCC25922	>50	50
E. coli BW25113	>50	25
E. coli BW25113 Δ*acrA**	>50	12.5 (6.25)
E. coli BW25113 Δ*tolC**	>50 (12.5)	6.25 (3.125)

Numbers in parentheses correspond to lugdunin concentrations leading to reduced, but still visible bacterial growth. *The efflux-deficient strains *E. coli* BW25113 Δ*acrA* (JW0452) and *E. coli* BW25113 Δ*tolC* (JW5503) were obtained from the Keio collection (3).

Table S2. Promoter assays using *B. subtilis* 1S34-based reporter constructs that signal stress on different bacterial metabolic pathways induced by antibiotic action. The performed promoter assays were either liquid-based (firefly luciferase reporter gene fusions) or agar-based (beta-galactosidase reporter gene fusions), as previously described (4-6).

	**Induction of promoter (targeted metabolic pathway)**				
**Antibiotic**	*ypuA* (cell envelope)	*liaI* (cell envelope)	*yorB* (DNA)	*helD/yvgS* (RNA)	*bmrC/yheI* (protein)
Lugdunin	-	-	-	-	-
CCCP	-	-	-	-	-
Valinomycin	-	-	n.d.	n.d.	n.d.
Gramicidin A	-	-	n.d.	n.d.	-
Gramicidin S	(+)	+	n.d.	n.d.	n.d.
Nisin	(+)	+	-	-	-
Daptomycin	+	+	-	-	-
Vancomycin	+	+	-	-	-
Ciprofloxacin	-	-	+	-	-
Rifampicin	-	-	-	+	-
Chloramphenicol	-	-	-	-	+

+, reporter induction; (+), weak reporter induction; -, no induction; n.d., not determined

References

1. Borst-Pauwels GWFH. 1976. Discrimination criteria for apparent two-site transport models. J Theor Biol 56:191-204.
2. Mendes SS, Marques J, Mesterházy E, Straetener J, Arts M, Pissarro T, Reginold J, Berscheid A, Bornikoel J, Kluj RM, Mayer C, Oesterhelt F, Friães S, Royo B, Schneider T, Brötz-Oesterhelt H, Romão CC, Saraiva LM. 2022. Synergetic Antimicrobial Activity and Mechanism of Clotrimazole-Linked CO-Releasing Molecules. ACS Bio Med Chem Au 2:419-436.
3. Baba T, Ara T, Hasegawa M, Takai Y, Okumura Y, Baba M, Datsenko KA, Tomita M, Wanner BL, Mori H. 2006. Construction of *Escherichia coli* K-12 in-frame, single-gene knockout mutants: the Keio collection. Molecular Systems Biology 2:1-11.
4. Wex KW, Saur JS, Handel F, Ortlieb N, Mokeev V, Kulik A, Niedermeyer THJ, Mast Y, Grond S, Berscheid A, Brötz-Oesterhelt H. 2021. Bioreporters for direct mode of action-informed screening of antibiotic producer strains. Cell Chem Biol 28:1242-1252.e4.
5. Urban A, Eckermann S, Fast B, Metzger S, Gehling M, Ziegelbauer K, Rübsamen-Waigmann H, Freiberg C. 2007. Novel whole-cell antibiotic biosensors for compound discovery. Applied and Environmental Microbiology 73:6436-6443.
6. Wenzel M, Chiriac AI, Otto A, Zweytick D, May C, Schumacher C, Gust R, Albada HB, Penkova M, Krämer U, Erdmann R, Metzler-Nolte N, Straus SK, Bremer E, Becher D, Brötz-Oesterhelt H, Sahl H-G, Bandow JE. 2014. Small cationic antimicrobial peptides delocalize peripheral membrane proteins. Proceedings of the National Academy of Sciences of the United States of America 111:E1409-18.
